# Dietary Nitrate Supplementation and Exercise Performance in Athletes: A Systematic Review and Meta-Analysis of Randomised Controlled Trials

**DOI:** 10.3390/sports14100466

**Published:** 2026-10-20

**Authors:** Yiqing Xie, Yemin Han, Qing Du, Kai Xu

**Affiliations:** 1 School of Sport, Exercise and Health Sciences, Loughborough University, Loughborough, Leicestershire LE11 3TU, UK; 2221151070@sus.edu.cn; 2 School of Athletic Performance, Shanghai University of Sport, Shanghai 200438, China; 3 School of Medical and Health Sciences, Centre for Human Performance, Edith Cowan University, Joondalup 6027, Australia

**Keywords:** dietary supplements, dietary nitrate, nitric oxide, exercise performance, physiological responses, randomized controlled trial

## Abstract

**Background/Objectives**: Dietary nitrate supplementation improves exercise performance in recreationally active individuals, but efficacy in athletes remains debated. This meta-analysis evaluated the effects of dietary nitrate supplementation on six exercise performance and physiological/perceptual outcomes—peak power (PP), mean power (MP), maximal oxygen uptake (VO_2_max), heart rate (HR), blood lactate (LAC), and rating of perceived exertion (RPE)—in athletes across diverse sports and protocols. **Methods**: Forty-five randomized, placebo-controlled trials involving 659 healthy athletes (Tier 2 or higher) were included. Random-effects meta-analyses calculated pooled Hedges’ g. Subgroup, sensitivity, and publication bias analyses were conducted. **Results**: No statistically significant overall effect was found for any of the six outcomes (all *p* > 0.05). In exploratory subgroup analyses, PP showed a positive effect in male-only studies (g = 0.29, 95% CI 0.02 to 0.56, *p* = 0.04), but the interaction with mixed-sex studies was not significant (*p* = 0.11). Beetroot and nitrate salts did not differ in VO_2_max. And trim-and-fill imputed five studies, reducing this estimate to zero. Subgroup analyses by sport type and exercise type (endurance, intermittent team sports, strength/power) revealed no significant differences. **Conclusions**: Dietary nitrate supplementation produced no statistically significant pooled ergogenic effects in athletes. Any potential benefit appears small and context-dependent, and the possible role of vascular endothelial function in high-performance athletes warrants further investigation. The absence of statistically significant effects should not be regarded as definitive evidence of no effect.

## 1. Introduction

Dietary nitrate supplementation, often provided as beetroot juice (BRJ) or inorganic nitrate salts, has become one of the most widely used ergogenic aids for aerobic endurance exercise, underpinned by a well-characterised physiological pathway: inorganic nitrate (NO_3_^−^) is reduced to nitrite (NO_2_^−^) by oral commensal bacteria, and subsequently to nitric oxide (NO) in hypoxic or acidic tissues, improving mitochondrial oxidative efficiency, skeletal muscle blood flow, and fast-twitch muscle contractility [1,2]. These mechanisms have translated to consistent, small-to-moderate performance benefits in recreationally active individuals and non-athletes, particularly during prolonged, submaximal or open-ended exercise tasks [3].

The efficacy of dietary nitrate supplementation in elite or well-trained athletes, however, remains more contentious. A growing body of evidence suggests that the ergogenic effect of nitrate diminishes with increasing aerobic fitness: a previous meta-analysis reported a significant effect size (ES) of 0.25 (95% CI 0.11 to 0.38) for nitrate in non-athletes, but no significant effect in athletes (ES = 0.04, 95% CI −0.05 to 0.15) [3]. Another study further narrowed this relationship, finding that time-trial performance improvements were restricted to less-trained individuals with a VO_2_max < 55 mL/kg/min, with no benefit in higher-fitness groups [4]. This pattern is supported by individual studies in elite populations: one trial found no improvement in 1500-m run performance in elite middle-distance runners [5]; another reported no change in VO_2_ kinetics or endurance in elite cyclists [6]; and a third observed no benefit for well-trained runners during altitude time trials [7]. A viewpoint article formalised this debate, prompting multiple commentaries proposing mechanistic explanations for the attenuated response in elite athletes: higher baseline NO bioavailability in trained individuals may blunt exogenous nitrate effects [8,9], non-site-specific NO production may divert blood flow to non-prioritised vascular beds during maximal exertion near cardiac output limits [10], and individual variability in oral nitrate reductase activity may create “responders” and “non-responders” that obscure group-level effects [11]. A tentative consensus has thus emerged that dietary nitrate is unlikely to produce meaningful performance gains in elite athletes, though small benefits may persist in specific contexts (e.g., short-burst intermittent exercise, altitude, or power-oriented events [12,13]).

Despite this progress, existing meta-analyses have several limitations that constrain firm conclusions about dietary nitrate supplementation’s effects on athlete exercise performance and physiological responses. This is well illustrated by several very recent systematic reviews with meta-analyses that directly address overlapping outcomes. A recent meta-analysis pooling 38 trials found a small improvement in VO_2_max/VO_2_peak after administration of beetroot juice (SMD = 0.24, 95% CI 0.11 to 0.37), but this effect was not significant in highly trained athletes (SMD = 0.07, 95% CI −0.09 to 0.23) [14]. Another recent meta-analysis reported significant improvements in high-intensity interval sprint performance (SMD = 0.38, 95% CI 0.17 to 0.59), mean power output (SMD = 0.43, 95% CI 0.21 to 0.64), and VO_2_max (SMD = 0.30, 95% CI 0.05 to 0.55) [15]. A third recent systematic review and meta-analysis, however, found no significant effects on peak power, mean power, 10-m or 20-m sprint performance, perceived exertion, heart rate, or blood lactate during single or repeated sprints, despite observing an improvement in time to peak power (SMD = −0.92, 95% CI −1.29 to −0.54) and handgrip strength (SMD = 0.40, 95% CI 0.05 to 0.74) [16]. These divergent findings underscore the extent to which outcome selection, participant fitness, exercise protocols, and supplementation strategies can influence meta-analytic conclusions. First, most reviews focus narrowly on a single performance outcome (e.g., time-trial completion time [17]; short-duration high-intensity power [18]; resistance exercise [19]) or a single sport (e.g., combat sports [20]; endurance cyclic sports [21]), with few synthesising the physiological and perceptual markers that underlie exercise tolerance and performance. One review noted contradictory findings for dietary nitrate and VO_2_max in athletes [22], yet no meta-analysis has comprehensively aggregated PP, MP, VO_2_max, HR, LAC, and RPE—six of the most widely used indices of exercise performance and acute physiological/perceptual responses to exercise—to disentangle whether dietary nitrate produces physiological improvements that fail to translate to performance, or no meaningful effects at any level. Second, excessive stratification of existing reviews may dilute detectable signals. For example, one study found that nitrate supplementation only improved PP during 24 × 6-s sprints with 24-s recovery, with no effect during longer intervals (730-s, 6 × 60-s) [23]; a meta-analysis that pools all “sprint” protocols would miss this specific, small effect. Most current reviews further separate acute vs. chronic supplementation, or individual sport modalities, leaving each with limited statistical power to detect the modest effects typical of nitrate research. Third, few previous meta-analyses have rigorously addressed small-study bias, despite evidence that positive small trials are over-represented in the sports nutrition literature. Moreover, the inconsistent findings among these recent meta-analyses may be explained by differences in participant eligibility (e.g., healthy adults of mixed training status vs. trained athletes), exercise protocols (e.g., graded incremental tests vs. Wingate vs. repeated sprints), supplementation strategies (e.g., acute single doses vs. multi-day loading; dose 6.4–12.8 mmol; juice source/brand), outcome definitions (e.g., VO_2_max vs. VO_2_peak; mean power vs. peak power vs. time to peak power), and analytical approaches (e.g., choice of effect measure, random-effects assumptions, treatment of small-study bias).

To address these gaps and to resolve these inconsistencies within a single athlete-focused dataset, the present meta-analysis aggregated 45 randomised, placebo-controlled trials across multiple athlete populations (cycling, running, kayaking, team sports) and testing modalities (laboratory ergometry, on-water/track performance, incremental and time-trial protocols) to comprehensively evaluate nitrate supplementation effects on the six core outcomes (PP, MP, VO_2_max, HR, LAC, RPE). These recent reviews each focused on one or two outcome families and included mixed-fitness populations. In contrast, the present review is restricted to trained athletes and simultaneously harmonises six outcome domains using consistent inclusion criteria, standardised outcome definitions, and a unified analytical framework, allowing direct comparison of performance, physiological, and perceptual effects [14,15,16]. It also incorporates both beetroot juice and inorganic nitrate salts, both acute and chronic supplementation protocols, and—where data permitted—crossover and parallel designs as moderator variables, and systematically addresses small-study bias for every outcome. These outcomes were selected because they represent commonly used indices of exercise performance and acute physiological/perceptual responses in athlete testing across a range of exercise modalities; they are not confined to strictly aerobic capacity measures. Subgroup analyses by sex, sport type, and testing measure were conducted to explore sources of heterogeneity without over-stratifying the sample, and sensitivity analyses and publication bias assessments were performed to verify robustness. This approach balances the need to avoid diluting small, context-specific effects with the need for a holistic assessment of dietary nitrate’s impact on athlete exercise performance and physiological/perceptual responses.

## 2. Materials and Methods

### 2.1. Systematic Review Protocol Registration

This study was conducted following the Cochrane Handbook for Systematic Reviews of Interventions (Version 6.0) guidelines [24] and adhered to the 2020 Preferred Reporting Items for Systematic Reviews and Meta-Analyses (PRISMA) statement [25]. The protocol was registered on the Open Science Framework (https://osf.io/8stk7/overview (accessed on 30 June 2026)). The initial literature search was completed on 6 May 2026, and an updated search was re-run on 17 September 2026, before resubmission. Because the protocol was registered on 30 June 2026, after the initial search had already been completed, this registration is retrospective rather than prospective. All analyses described in this manuscript were pre-specified in the registered protocol, with two exceptions: subgroup analyses comparing beetroot juice vs. inorganic nitrate salts and crossover vs. parallel designs, which were added post hoc during the revision process in response to reviewer feedback.

### 2.2. Eligibility Criteria

Studies eligibility followed a PICOS approach (Table 1). The eligibility criteria are summarized in Table 1.

### 2.3. Information Sources and Search Strategy

The following databases were searched from inception to 17 September 2026: PubMed/MEDLINE, SCOPUS, CINAHL (Cumulative Index to Nursing and Allied Health Literature), SPORTDiscus, and Web of Science. No year limits were applied. The original search was conducted on 6 May 2026, and search alerts were monitored thereafter to identify any new publications. A complete re-run of the search across all databases was conducted on 17 September 2026, to ensure the review captured all eligible studies published up to that date. Secondary searching included: (a) screening reference lists of all included studies and relevant review articles; (b) forward citation tracking of included studies via Google Scholar; and (c) monitoring search alerts after 1 July 2026, with a final comprehensive update performed on 17 September 2026 as described above. To minimize publication bias and ensure the inclusion of studies with complete and peer-reviewed methodologies, the search was limited to published, peer-reviewed journal articles. Grey literature sources, such as conference proceedings and theses, were not actively searched or included.

### 2.4. Study Selection

Duplicate records were first removed using EndNote reference manager (Version X9.0.3; Clarivate Analytics, Philadelphia, PA, USA). Two authors (YQX and YMH) then independently screened titles and abstracts using the Rayyan web application (https://rayyan.ai (accessed on 6 May 2026); Rayyan Systems Inc., Cambridge, MA, USA) to assess eligibility based on the inclusion criteria. To avoid bias, the process was blinded regarding author information. Subsequently, the same two authors (YQX and YMH) independently assessed the full texts of potentially eligible studies. Any disagreements regarding eligibility at any stage were resolved through discussion, with arbitration by a third reviewer (KX) if necessary.

### 2.5. Data Extraction

The following data were extracted from included studies into a standardized Excel spreadsheet (Microsoft Corporation, Redmond, WA, USA): (1) study design and identification information; (2) adherence, study duration, groups, and outcome measures used; (3) sample size; (4) participant characteristics (age, sex, sport, sports experience and/or training load, VO_2_max); (5) supplementation protocol details, including nitrate dose (mmol/day), timing of the last dose relative to exercise (hours), exercise protocol, and principal performance measures; and (6) pre- and post-intervention means, standard deviations (SDs), and raw mean changes with SDs of changes for both supplement and placebo groups for relevant outcomes. If data were insufficiently reported, the corresponding authors of those studies were contacted via email. When authors did not report or provide data, WebPlotDigitizer software (Version 4.1; https://automeris.io/WebPlotDigitizer/ (accessed on 8 June 2026); Ankit Rohatgi, Pacifica, CA, USA) was used to extract data from figures. Data extraction was performed independently by three authors (YQX, YMH, and QD) using a pre-piloted form tested on five randomly selected studies and subsequently modified. Extracted data were cross-checked among authors, and any discrepancies were resolved through discussion and consensus, or by a fourth reviewer (KX).

Given the breadth of outcomes included, we deliberately refrained from classifying PP, MP, VO_2_max, HR, LAC, and RPE under a single physiological construct (e.g., “aerobic capacity”). Instead, these outcomes are reported as exercise performance metrics (PP, MP), maximal cardiorespiratory capacity (VO_2_max), and acute physiological (HR, LAC) or perceptual (RPE) responses to standardized exercise protocols.

We conducted subgroup analyses to examine the effects of six categorical moderators: supplement source, biological sex, sport type, exercise type, testing measure, and design type. Supplement source was classified as beetroot juice, inorganic nitrate salts, or other nitrate-rich vegetables (e.g., spinach homogenate), with the primary comparison being beetroot juice versus inorganic nitrate salts. Biological sex was classified as male, female, or mixed (studies that included both sexes). Sport type was categorized based on the athletic disciplines reported in the primary studies. Examples include soccer, cycling, running, rowing, kayaking, cross-country skiing, swimming, triathlon, fencing, and others. Exercise type was classified as endurance, intermittent team sports, strength/power, or other, based on established physiological frameworks: intermittent team sports followed the intermittent high-intensity activity patterns described elsewhere [30]; endurance sports followed the established aerobic performance model [31]; strength/power sports applied the neuromuscular power development framework [32]; and other/composite sports were classified according to the skill-based demands outlined in previous work [33], with overall guidance from the training periodization principles in the literature [34]. Testing measure referred to the specific exercise test protocol used to evaluate exercise performance and physiological/perceptual responses (Wingate anaerobic test, time-trial over various distances, submaximal constant-load cycling, incremental ramp test to exhaustion, etc.). Design type was classified as crossover or parallel. Each subgroup analysis was performed only when at least two levels contained at least three studies. These moderators were used in mixed-effects meta-regression models to explore whether the effect of nitrate supplementation varied by study or participant characteristics.

### 2.6. Risk of Bias Assessment

To appraise the completeness of study methodology and reporting, we used a modified PEDro scale [35]. This 10-item tool (total score range 0–20) evaluates the following criteria: explicit inclusion criteria; random allocation of participants; clear definition of the intervention; baseline similarity between groups; presence of a control condition; clear definition of outcome variables; practical usefulness of the assessments and of the intervention duration; appropriateness of between-group statistical analyses; and reporting of point estimates and variability. Each item was rated as 0 (clearly no), 1 (maybe), or 2 (clearly yes). A study was considered eligible only if its total score reached the pre-specified threshold of ≥16; otherwise, together with studies whose outcome data could not be retrieved, it was excluded during screening (*n* = 10), as indicated in the PRISMA flow diagram. Importantly, this screening tool was used exclusively to determine initial eligibility; the final risk-of-bias appraisal of every included study was performed with the Cochrane Risk of Bias 2 (RoB 2) tool described below. Before conducting the assessments, the three assessing authors (YQX, YMH, and KX) discussed the modified PEDro scale and the RoB 2 tool to ensure a uniform interpretation, after which all evaluations were performed independently. Any disagreements were resolved through discussion.

Risk of bias was assessed using the Cochrane Risk of Bias 2 tool (RoB 2) for randomized trials [36]. Because most included trials assigned individuals (or, where applicable, used randomized crossover designs), the five RoB 2 domains were applied: (1) bias arising from the randomization process; (2) bias due to deviations from intended interventions; (3) bias due to missing outcome data; (4) bias in measurement of the outcome; and (5) bias in selection of the reported result. For crossover trials, domain 2 was interpreted accounting for potential period/carry-over and the adequacy of washout periods.

Given that nitrate supplementation often cannot be fully blinded because of taste, colour, or mild physiological cues, domain 2 was retained in full; signalling questions regarding blinding of participants and personnel were rated as high risk or some concerns where credible blinding was unlikely or not verified, and we additionally recorded whether trials used nitrate-depleted placebo matched for appearance/taste, standardized instructions, and independent outcome assessment to reduce performance and detection bias. Allocation generation and concealment (domain 1) were rated strictly; studies that used quasi-random methods (alternation, date of enrolment, etc.) were rated as high risk in domain 1 and explored in sensitivity analyses.

To capture exercise- and supplement-specific sources of heterogeneity that are not formal RoB 2 domains but may affect internal validity, we also extracted/protocolled the following supplementary appraisal items (reported narratively/explored in subgroup or sensitivity analyses): (i) adherence/compliance monitoring (e.g., capsule counts, plasma/urine nitrate/NO_2_^−^ verification); (ii) standardization of training and physical activity during the supplementation window; (iii) familiarization with testing procedures; and (iv) control of dietary nitrate (e.g., low-nitrate diet instruction/check).

Final judgments per domain were made as low risk, some concerns, or high risk following the Cochrane algorithm [36]. Two reviewers (YQX and YMH) conducted the assessments independently; discrepancies were resolved by consensus or, when necessary, by a third reviewer (KX). For the overall study-level judgment, we followed the complete Cochrane RoB 2 algorithm, including the provision that multiple domains rated as some concerns may, in combination, warrant an overall high-risk judgment even when no single domain is high risk. Specifically, an overall judgment of low risk required all five domains to be low risk; an overall judgment of high risk was assigned when any single domain was high risk or when the reviewers judged that the number and nature of domains rated as some concerns substantially reduced confidence in the study’s results. In line with this provision, we a priori specified that studies with three or more domains simultaneously rated as some concerns would be classified as high risk, because the accumulation of concerns across domains such as random sequence generation, blinding, and selective outcome reporting would materially lower confidence in the reliability of the effect estimates. Disagreements were resolved by consensus or by a third reviewer.

### 2.7. Certainty Assessment

The certainty of evidence for each outcome was assessed using the Grading of Recommendations Assessment, Development and Evaluation (GRADE) approach with randomized trials as the starting point (rated as high certainty initially) [37]. Certainty was rated down across five domains: (1) risk of bias (based on RoB 2 judgments described in Section 2.6); (2) inconsistency (I^2^ > 50% and/or τ^2^ indicating substantial heterogeneity unexplained by subgroup analyses); (3) indirectness (differences between the available evidence and the review PICO—e.g., variation in athlete population [sport type, sex, training status], nitrate dose and timing and testing modality); (4) imprecision (confidence interval width crossing the minimally important difference, or total sample size < 200 per outcome per GRADE thresholds for continuous outcomes); and (5) publication bias (assessed via funnel plot asymmetry, Egger’s test, and trim-and-fill).

GRADE ratings were performed per outcome (PP, MP, VO_2_max, HR, LAC, RPE) for the comparison dietary nitrate supplementation vs. placebo. Two reviewers (YQX and YMH) conducted the assessments independently, with verification by a third reviewer (KX). The final certainty level (high, moderate, low, very low) was assigned per outcome by synthesizing judgments across the five domains. Results are presented in Summary of Findings tables (one per outcome domain) following GRADE guidance.

### 2.8. Statistical Analysis

All analyses were performed in R (Version 4.2.1; R Foundation for Statistical Computing, Vienna, Austria) [38] using the metafor [39] and meta [40] packages.

For each outcome, we calculated the between-group standardized mean difference (Hedges’ g [41]) comparing pre-to-post change scores between the nitrate supplementation (supplement) and placebo groups. Within-group change scores were computed assuming a pre–post correlation of r = 0.5 [42]. This default value is empirically justified: a large empirical assessment of within-group correlations across 123 studies found a median correlation of 0.59 (IQR 0.40–0.81), and imputing r = 0.5 yielded standard errors closest to those actually reported in the source studies [42]. Pooled overall effects were estimated with random-effects models using restricted maximum likelihood (REML [39]). Heterogeneity was quantified via τ^2^ (between-study variance), I^2^ (proportion of total variance due to true heterogeneity [43]), and the Q test [41]. Where the REML estimate of τ^2^ was truncated at zero, I^2^ was reported as 0.00%; this indicates that no between-study variance was detected given the available statistical power, and does not constitute proof of exact homogeneity. Both τ^2^ and I^2^ are reported for each outcome in the Results.

To ensure statistical independence, each study contributed only one effect size per outcome. When multiple measurements from the same cohort were eligible for an outcome (e.g., different exercise intensities, split distances, repeated test bouts, or sampling time points), a pre-defined two-stage selection procedure was applied. In the first stage, the measurement that most directly matched the pre-specified outcome definition was selected: for peak power, the maximal short-duration effort; for mean power, the sustained or mechanically stable phase of the trial; for VO_2_max, the highest oxygen uptake value from a graded test or, when only submaximal data were reported, the highest submaximal intensity with complete steady-state data; for heart rate, the steady-state value at the highest comparable exercise intensity; and for blood lactate and RPE, the value closest to exercise cessation or to the highest recorded stage. In the second stage, if more than one measurement remained, we selected the value least susceptible to familiarisation, fatigue, or incomplete recording: for repeated-bout protocols, the mean of the two most stable trials; for split-distance protocols, the segment(s) whose metabolic demand best matched the outcome of interest; and for multiple exercise intensities, the intensity range that provided the most complete and representative data. For studies comparing multiple nitrate doses, the dose closest to 6.2–8.4 mmol NO_3_^−^ was chosen. Finally, if multiple effect sizes were still equally qualified, the dataset with the largest sample size and the most conservative estimate was selected. Studies presenting data from independent subgroups (e.g., males and females reported separately) contributed separate effect sizes. All specific selection decisions are documented in Supplementary Table S3, and sensitivity analyses based on alternative selections confirmed that the direction and significance of the pooled estimates were unchanged.

Analysis of crossover trials. Both crossover and parallel-group designs were included. Crossover trials present a methodological challenge for meta-analysis because the precision estimates that properly account for the paired nature of the design are often unavailable from primary study reports [44]. Within the framework of Morris and DeShon [45], a crossover trial can yield two types of standardized effect sizes: a “repeated-measures” effect size that incorporates the within-participant correlation and an “independent-groups” effect size equivalent to that obtained from a parallel design. These two are mathematically related through the Pearson correlation between the paired conditions [45]. For crossover trials, we adopted a conservative independent-groups approximation: the effect size and its sampling variance were calculated using the standard formulae for two independent groups (i.e., ignoring the within-participant pairing between the intervention and placebo conditions). This approach is defensible because the primary studies typically reported only the means and standard deviations of the change scores for each condition separately, without providing the individual-level paired data or the within-participant correlation between conditions [44]. The independent-groups approximation yields unbiased point estimates but overestimates the sampling variance (i.e., produces wider confidence intervals), making it a conservative choice that reduces the risk of Type I error [46]. To evaluate the impact of this approximation, we conducted an additional sensitivity analysis in which we assumed a range of plausible cross-condition correlations (r_cross = 0.30, 0.50, 0.70) between the change scores of the intervention and placebo conditions. When meta-analyzing paired data without access to the raw correlation, it is recommended to examine the robustness of conclusions by sensitivity analyses across a range of plausible correlation values [47]. Under each assumption, the variance of the paired difference was recalculated by adjusting the independent-groups variance to account for the implied covariance between the paired conditions. The pooled effect was then re-estimated using the adjusted variances. This sensitivity analysis confirmed that the overall results were robust to the choice of r_cross (see Section 3.5.3 and Supplementary Table S1). Across the entire manuscript, total participant counts refer to unique individuals only. In crossover trials, the same participants completed both the supplement and placebo conditions; therefore, the sample sizes shown for each condition in Table 2 were never summed, and the total N reflects unique participants. For the GRADE imprecision rating, the number of unique participants per outcome was used, rather than the combined SP + PLA values.

To explore potential sources of heterogeneity, we fitted mixed-effects meta-regression models [39] for six categorical moderators: supplement source, biological sex, sport type, exercise type, testing measure, and design type. Supplement source was classified as beetroot juice, inorganic nitrate salts, or other nitrate-rich vegetables (e.g., spinach homogenate). Because only one study used other nitrate-rich vegetables, this category was retained in the overall pooled analysis but excluded from the supplement-source subgroup comparison, which therefore focused on beetroot juice versus inorganic nitrate salts. Exercise type was classified as endurance, intermittent team sports, strength/power, or other, based on established physiological frameworks [30,31,32,33,34]. For each moderator, two specifications were compared: a separate-intercept model (mods = ~factor(x) − 1) to obtain level-specific pooled estimates and an interaction model (mods = ~factor(x)) to test whether effects differed across levels via the omnibus QM test (the multivariate analogue of the Q test for moderators). A significant omnibus test (QM *p* < 0.05) was taken to indicate a moderating effect. To avoid over-parameterisation, only moderator levels supported by ≥3 independent studies were retained for subgroup inference. Visualisations included forest plots (metafor), orchard plots (orchaRd [48]), funnel plots, and radial plots. Sensitivity analyses comprised leave-one-out diagnostics and two sets of correlation sensitivity analyses: (i) pre–post correlation, repeating the primary analysis with r = 0.3, 0.5, and 0.7 to assess robustness to the assumed pre–post correlation [42], and (ii) cross-condition correlation, used for crossover trials only, repeating the analysis with r_cross = 0.3, 0.5, and 0.7 as described above. Publication bias was assessed using: funnel-plot inspection; Egger’s regression test [49]; and the trim-and-fill procedure [39,50]. The final model output for each outcome reports Hedges’ g, 95% CI, *p*, τ^2^, I^2^, and the test of heterogeneity; subgroup/interaction results are reported with the QM χ^2^ and its *p*-value.

## 3. Results

### 3.1. Search Results

**Figure 1:**
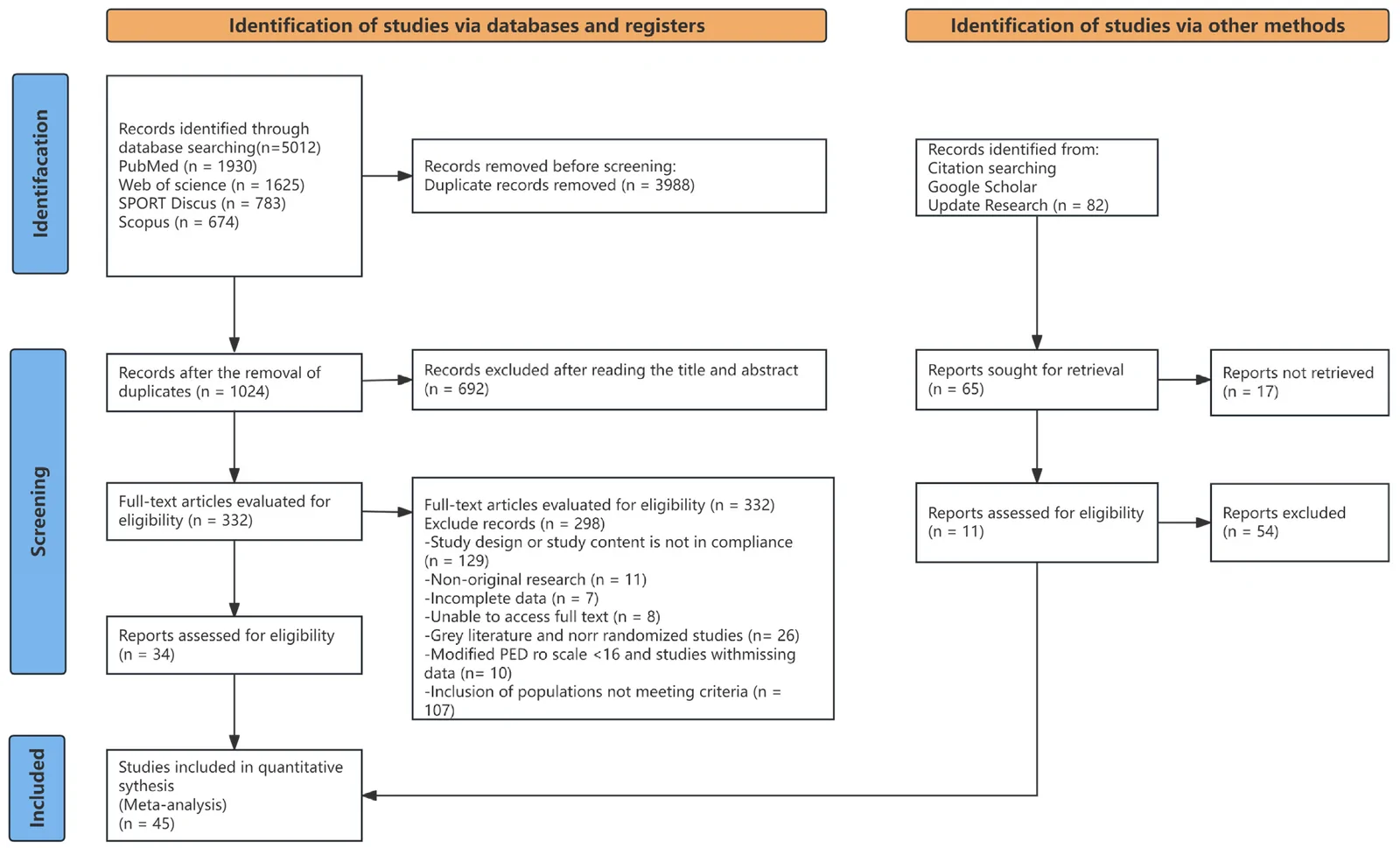
PRISMA flow diagram detailing the study inclusion process.

The initial search yielded 5012 records. Of these, 3988 duplicates were removed, resulting in 1024 unique records for title and abstract screening. The high proportion of duplicates (79.6%) reflects substantial overlap in the coverage of the databases searched (PubMed/MEDLINE, SCOPUS, CINAHL, SPORTDiscus, and Web of Science), which index many of the same journals and conference proceedings across multiple platforms. After screening titles and abstracts, 332 full-text documents were assessed for eligibility. Of these, 298 were excluded, and 34 studies met the inclusion criteria. Forward citation tracking and monitoring for newly published studies identified 11 additional studies, resulting in a total of 45 studies included in this review. Figure 1 illustrates the search and study selection process.

### 3.2. Study Characteristics

Among the 45 included studies, all were randomized controlled trials (either parallel-group or crossover designs), and 41 of 45 studies used a crossover design. The total number of participants across studies was 659 (556 males, 88 females, and 15 participants whose sex was not explicitly reported in the source data). Only three studies included females only, six included both male and female participants, and the remaining 36 studies included males only. Participants represented a wide range of sports, including cyclists, triathletes, runners, rowers, kayakers, cross-country skiers, swimmers, soccer players, ringette athletes, fencers, taekwondo athletes, CrossFit athletes, and hockey players, with training backgrounds ranging from recreational to elite international level.

The supplementation protocols varied substantially across studies. Nitrate supplementation or sodium/potassium nitrate was administered as a single acute dose in the majority of studies (*n* = 29), whereas 16 studies employed a multi-day loading strategy (3–8 days). The daily nitrate dose ranged from 4.0 to 19.35 mmol, with the most common doses being approximately 6.2–8.4 mmol (equivalent to 70–140 mL of concentrated BJ). The last dose was typically taken 2–3 h before exercise (range: 1.25–24 h). Placebo conditions consisted of nitrate-depleted BJ, blackcurrant juice, or inert capsules matched for appearance and taste.

Exercise testing protocols included time trials (e.g., 1 km, 4 km, 10 km, 20 km, 2000 m rowing), submaximal constant-load exercise (e.g., cycling at 45–70% VO_2_max, treadmill running at various intensities), incremental tests to exhaustion, Wingate anaerobic tests, repeated-sprint protocols, and sport-specific field tests. The principal outcome measures extracted were PP, MP, VO_2_max, HR, LAC, and RPE, which formed the basis for the meta-analysis.

All studies used free-living conditions without strict inpatient control, but most instructed participants to refrain from caffeine, alcohol, and vigorous exercise 24–48 h before testing, and to avoid antibacterial mouthwash during the supplementation period. The median intervention duration for chronic loading studies was 6 days (range 3–8 days), and the median washout period for crossover trials was 7 days (range 4–14 days).

### 3.3. Risk of Bias Assessment

**Figure 2:**
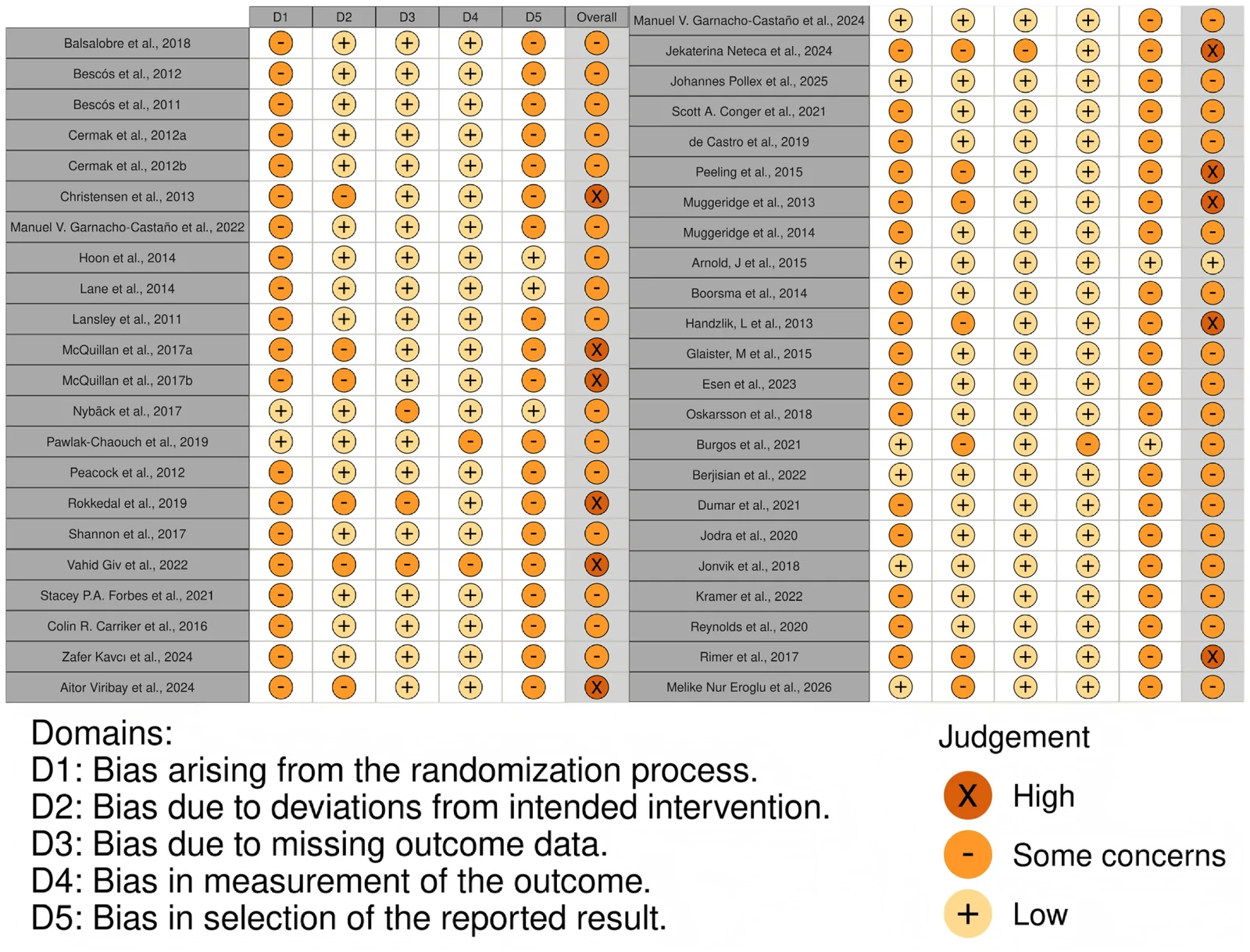
Risk of bias. References in Supplementary Files: Balsalobre et al., 2018 [51]; Bescós et al., 2012 [52]; Bescós et al., 2011 [53]; Cermak et al., 2012a [54]; Cermak et al., 2012b [55]; Christensen et al., 2013 [6]; Manuel V. Garnacho-Castaño et al., 2022 [56]; Hoon et al., 2014 [57]; Lane et al., 2014 [58]; Lansley et al., 2011 [59]; McQuillan et al., 2017a [60]; McQuillan et al., 2017b [61]; Nybäck et al., 2017 [62]; Pawlak-Chaouch et al., 2019 [63]; Peacock et al., 2012 [64]; Rokkedal et al., 2019 [65]; Shannon et al., 2017 [66]; Vahid Giv et al., 2022 [67]; Stacey P.A. Forbes et al., 2021 [68]; Colin R. Carriker et al., 2016 [69]; Zafer Kavcı et al., 2024 [70]; Aitor Viribay et al., 2024 [71]; Manuel V. Garnacho-Castaño et al., 2024 [72]; Jekaterina Neteca et al., 2024 [73]; Johannes Pollex et al., 2025 [74]; Scott A. Conger et al., 2021 [75]; de Castro et al., 2019 [76]; Peeling et al., 2015 [77]; Muggeridge et al., 2013 [78]; Muggeridge et al., 2014 [79]; Arnold, J et al., 2015 [7]; Boorsma et al., 2014 [5]; Handzlik, L et al., 2013 [80]; Glaister, M et al., 2015 [81]; Esen et al., 2023 [82]; Oskarsson et al., 2018 [83]; Burgos et al., 2021 [84]; Berjisian et al., 2022 [85]; Dumar et al., 2021 [86]; Jodra et al., 2020 [87]; Jonvik et al., 2018 [88]; Kramer et al., 2022 [89]; Reynolds et al., 2020 [90]; Rimer et al., 2017 [91]; Melike Nur Eroglu et al., 2026 [92].

For random sequence generation, 9 studies demonstrated a low risk of bias by reporting appropriate methods (e.g., computer-generated sequences, minimization software, or external researcher-generated randomization). The remaining 36 studies raised some concerns because the method of sequence generation was not described or was insufficiently reported. For blinding of participants and personnel (deviations from intended interventions), 32 studies were rated as low risk owing to adequate double-blinding with matched placebos, appropriate washout periods, and strict dietary/oral-hygiene controls. Thirteen studies raised some concerns because of incomplete reporting of placebo matching, lack of blinding checks, or omission of mouthwash restrictions. No studies were at high risk, as all included trials employed at least a placebo control and a randomized or counterbalanced design. Missing outcome data was adequately addressed in 41 studies (low risk), with no or minimal dropouts and clear reasons for exclusions. Four studies raised some concerns because of post-randomization exclusions without reporting group allocation or use of per-protocol analysis. Measurement of the outcome was at low risk in 42 studies, as these used objective, calibrated instruments (e.g., cycle ergometers, treadmills, gas analyzers, timing gates, dynamometers) under double-blind conditions, with standardized protocols and automated data recording. Three studies raised some concerns due to methodological limitations that could introduce assessor bias: one single-blind crossover study used an exhaustion endpoint (number of repetitions) that may be influenced by participants’ awareness of allocation; another open-label randomized controlled trial employed a field-based performance test (Bangsbo test) with subjective assessment and lacked blinding of outcome assessors; and a third double-blind study relied on manual counting and subjective validation of sit-up repetitions without independent verification or video recording. No studies were judged as high risk for this domain. Selective outcome reporting was assessed as low risk in only 5 studies that were prospectively registered and clearly specified a primary outcome. The remaining 40 studies raised some concerns because no preregistration was mentioned and multiple outcomes were reported without a pre-specified primary endpoint. After applying the complete RoB 2 algorithm, including the compounding-concerns provision, the overall risk of bias was low in 1 study, some concerns in 33 studies, and high in 11 studies. The 11 studies rated as high risk all had three or more domains simultaneously judged as some concerns; in these studies, combined doubts regarding random sequence generation, blinding or allocation concealment, and outcome reporting collectively weakened confidence in the reliability of their findings. The majority of studies demonstrated acceptable methodological rigor in blinding, dietary control, and objective measurements, but deficiencies in randomization reporting and lack of preregistration were common. A study-by-study breakdown of domain-level and overall risk-of-bias judgments is provided in Figure 2 and Supplementary Table S6.

### 3.4. Certainty Assessment

The overall certainty of the evidence was evaluated using the GRADE framework. As all included studies were randomized controlled trials, the initial certainty rating for all outcomes was high. However, the certainty for all outcomes was downgraded by one level because the overall risk of bias across studies was rated as serious: applying the complete Cochrane RoB 2 algorithm, 1 study was at low risk, 33 at some concerns, and 11 at high risk. For PP and MP, the certainty was downgraded an additional level because Egger’s regression test indicated potential small-study effects (PP: z = 3.03, *p* = 0.01; MP: z = 2.33, *p* = 0.03), and trim-and-fill analysis for PP imputed five missing studies, attenuating the pooled effect toward null; therefore, the publication-bias domain was rated as serious. While Egger’s test can be confounded by heterogeneity at small k, the downgrade for PP and MP was additionally supported by the accompanying trim-and-fill results. For VO_2_max and HR, no additional downgrades were applied because Egger’s test was non-significant and trim-and-fill did not materially alter the pooled estimates. For LAC and RPE, the certainty was downgraded two additional levels: one for serious inconsistency, reflecting the considerable heterogeneity across studies, and one for serious imprecision, owing to wide confidence intervals that crossed zero. For this imprecision rating, we used the total number of unique participants per outcome, not the combined supplement and placebo sample sizes. Consequently, the final overall certainty of evidence is moderate for VO_2_max and HR, low for PP and MP, and very low for LAC and RPE. The detailed certainty assessments for each outcome are presented in Table 2.

### 3.5. Main Results

#### 3.5.1. Overall Effects of Dietary Nitrate Supplementation on Exercise Performance and Physiological/Perceptual Responses

**Figure 3:**
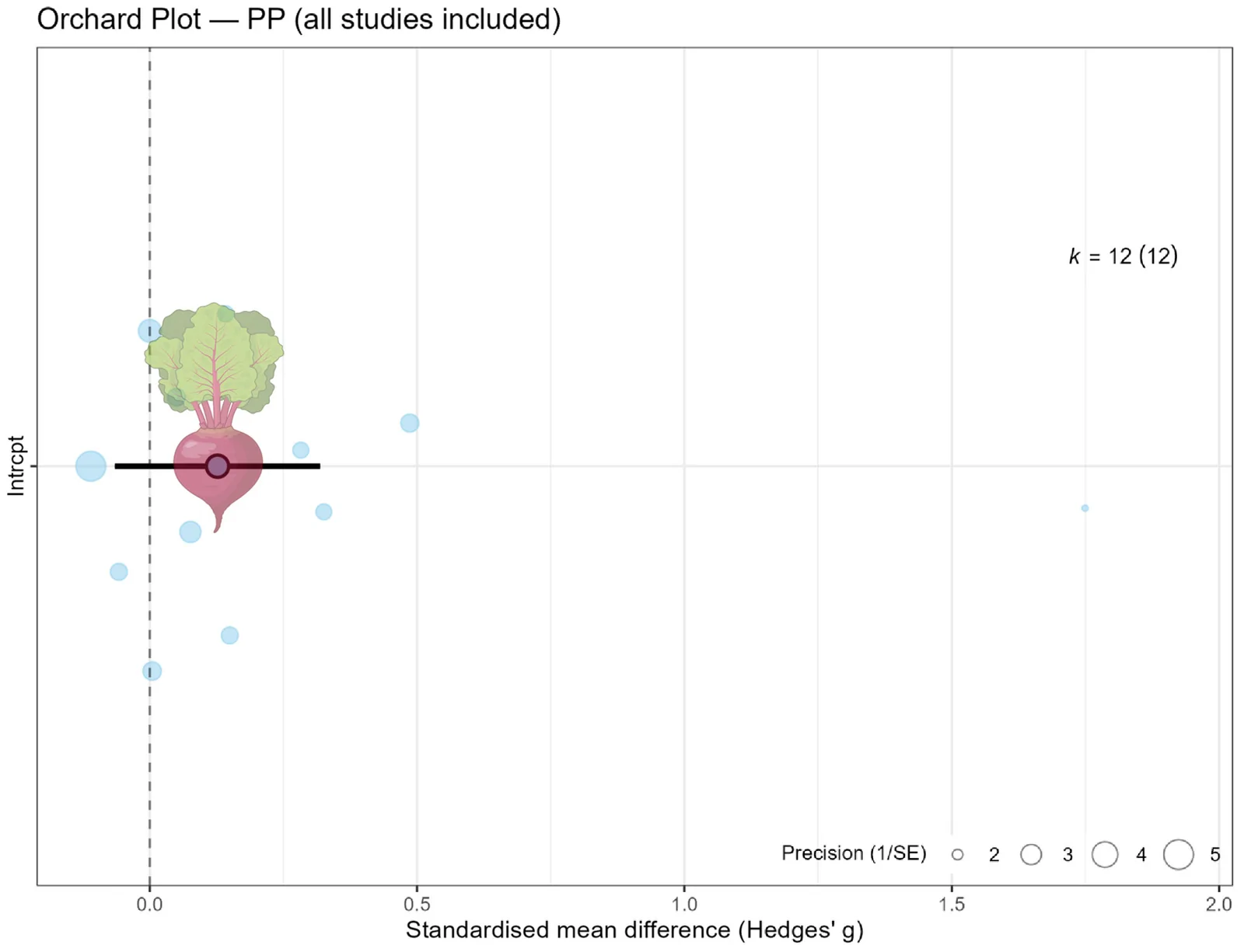
Orchard Plot of Peak Power (Beetroot Juice vs. Placebo).

**Figure 4:**
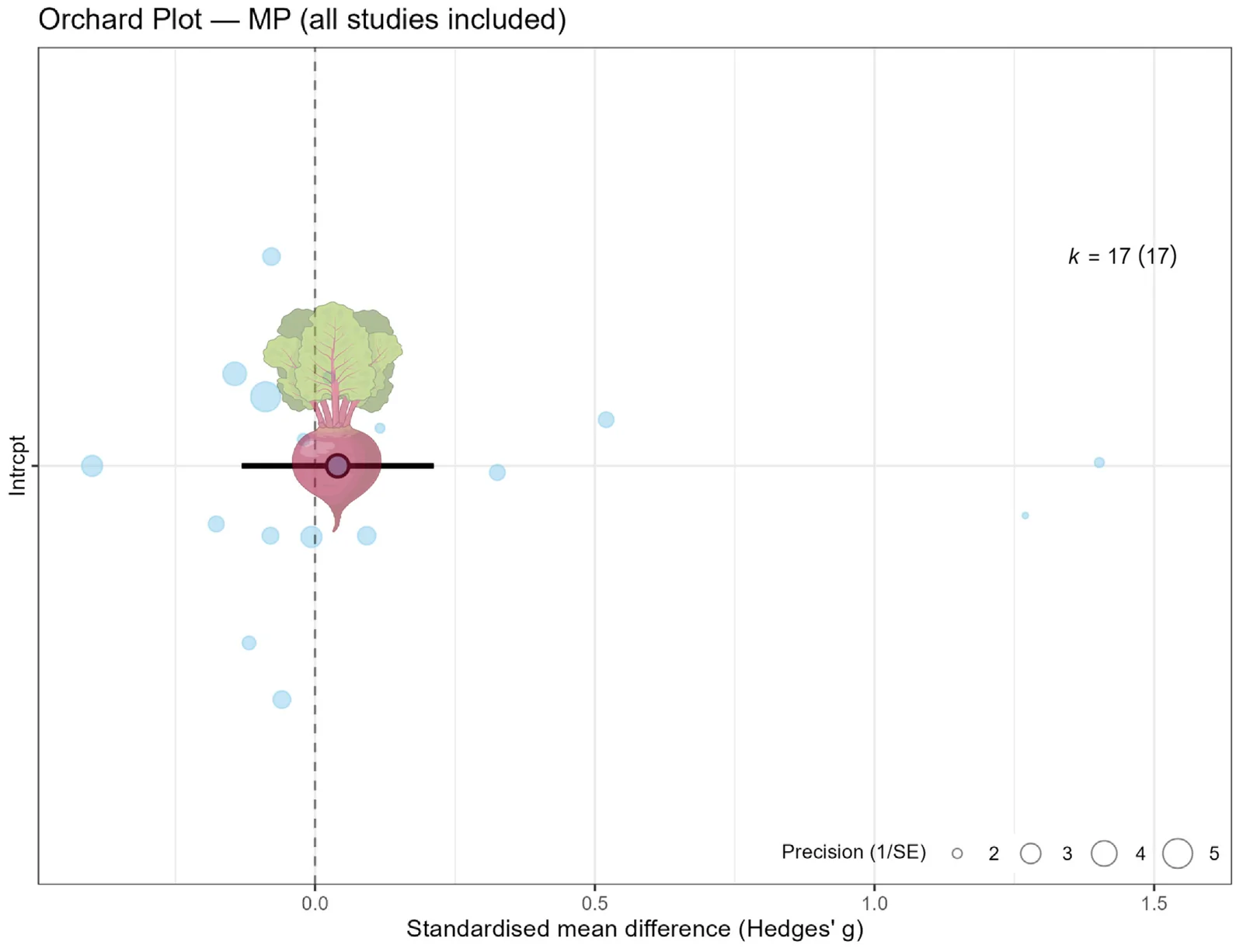
Orchard Plot of Mean Power (Beetroot Juice vs. Placebo).

**Figure 5:**
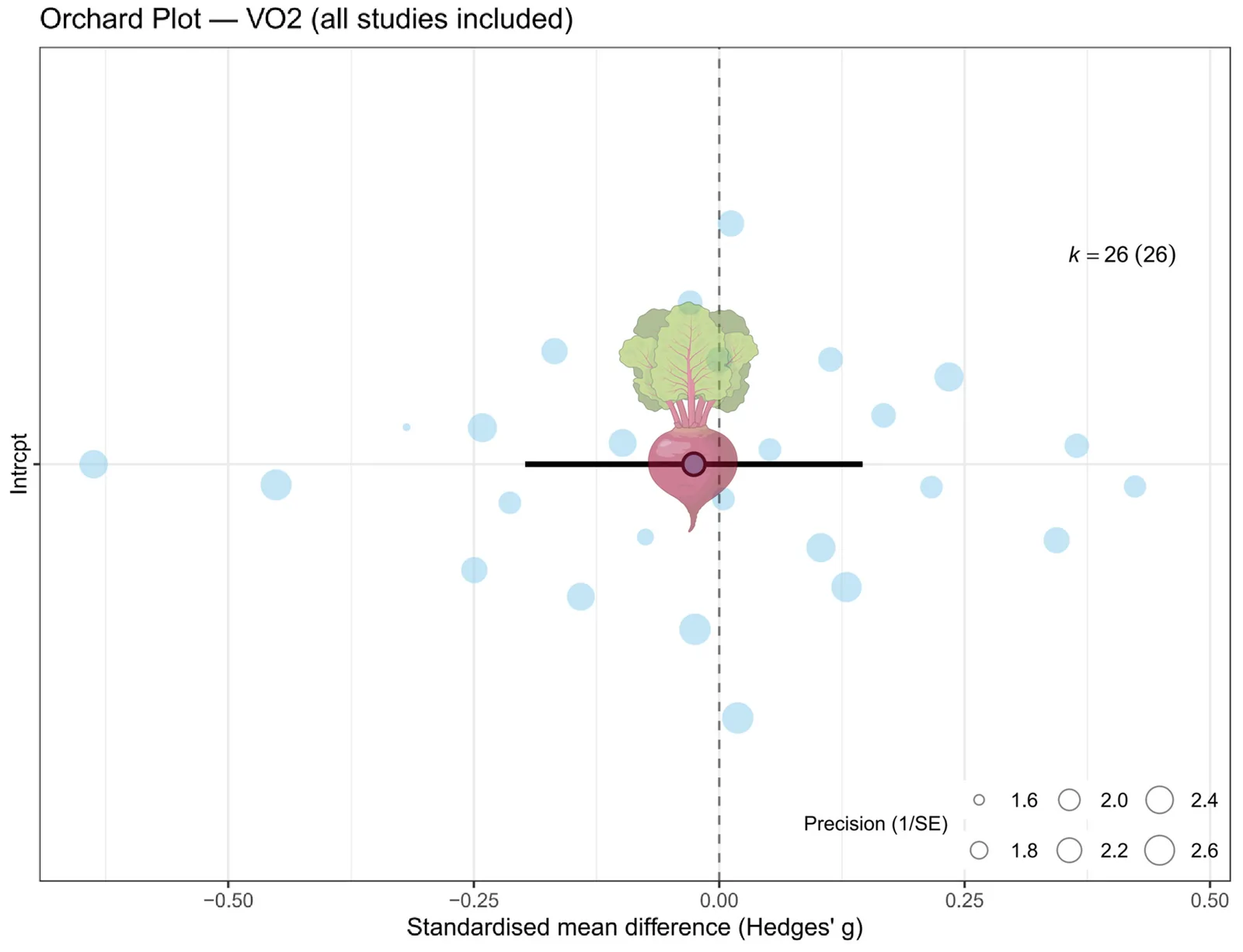
Orchard Plot of Maximal Oxygen Uptake (Beetroot Juice vs. Placebo).

**Figure 6:**
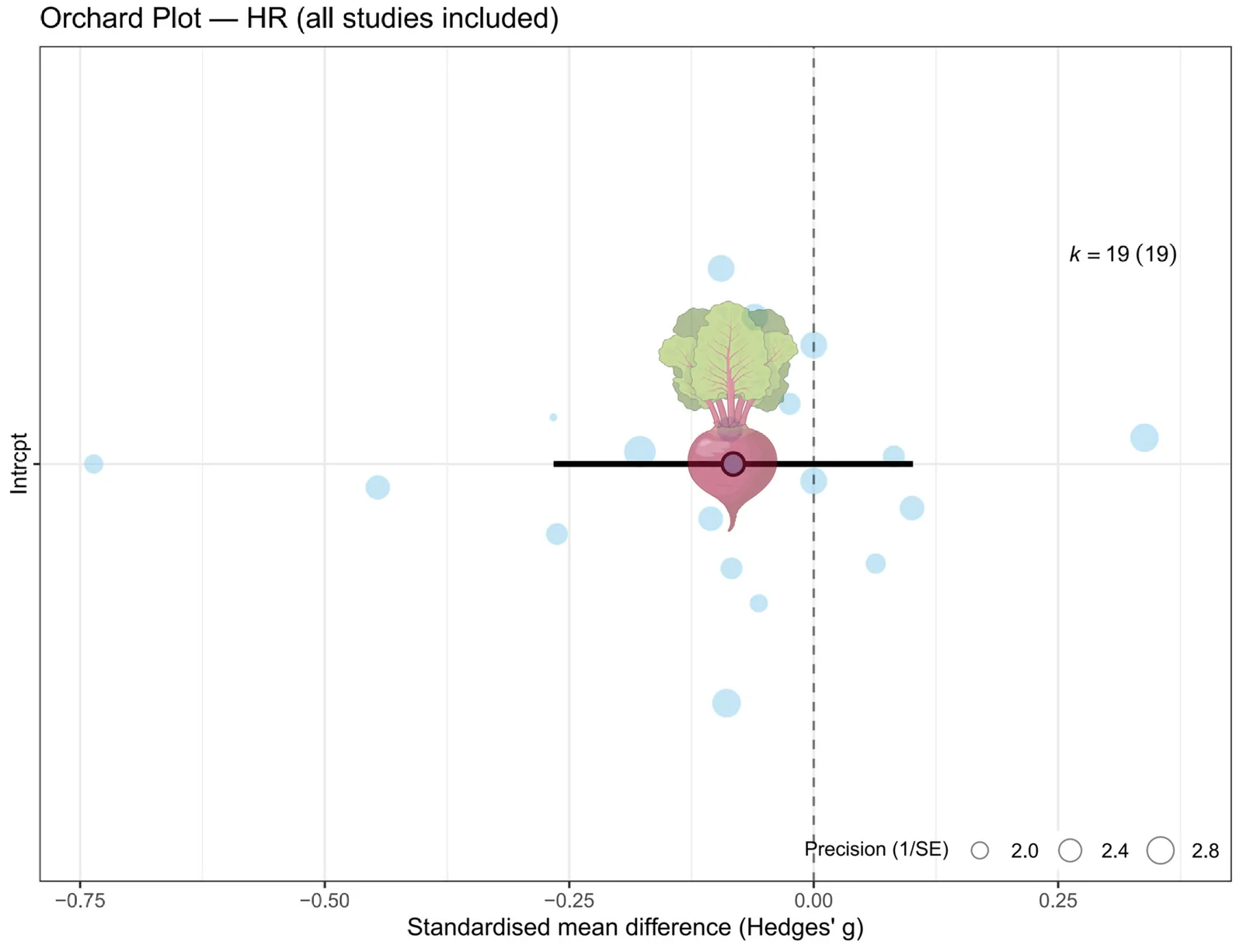
Orchard Plot of Heart Rate (Beetroot Juice vs. Placebo).

**Figure 7:**
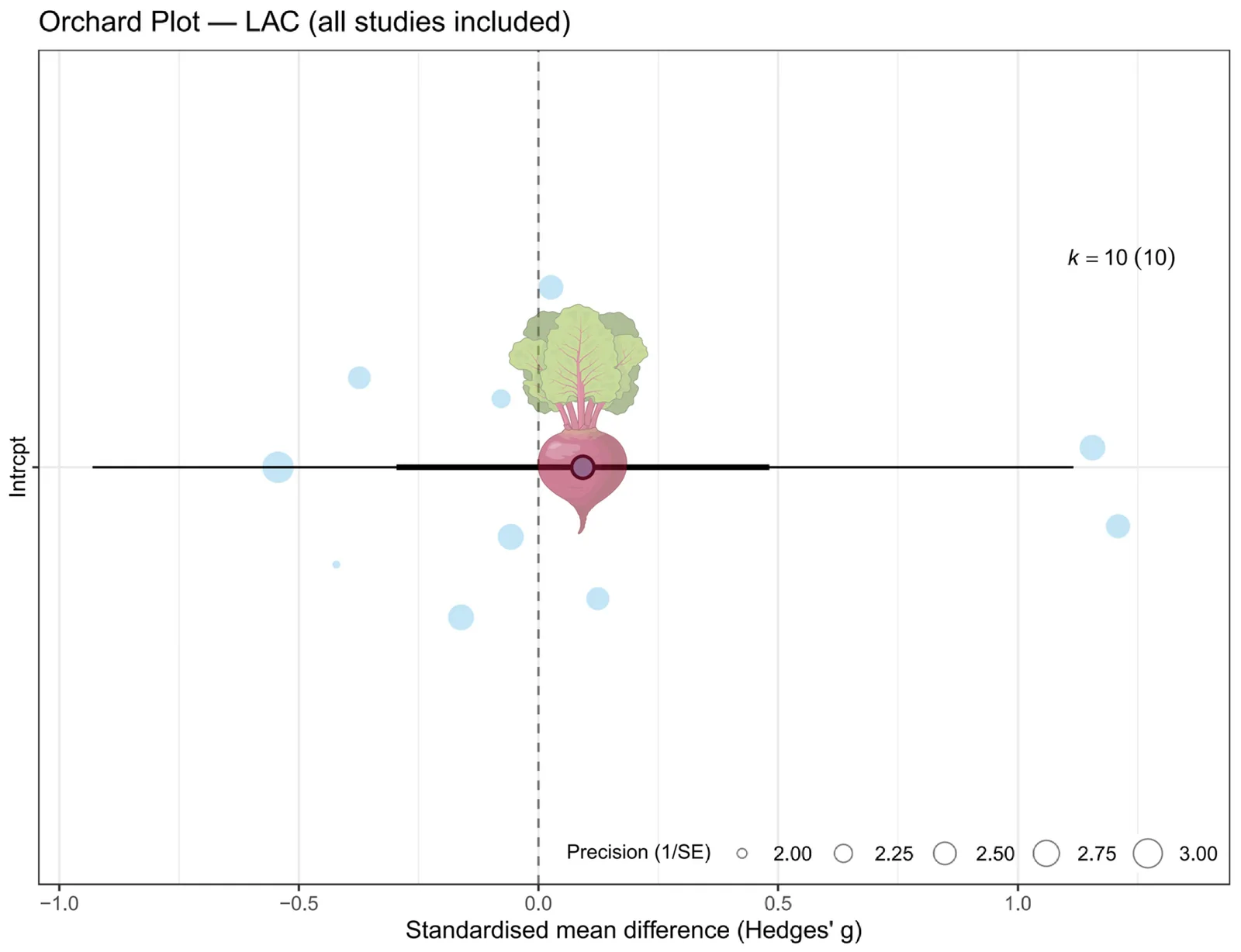
Orchard Plot of Blood Lactate Concentration (Beetroot Juice vs. Placebo).

**Figure 8:**
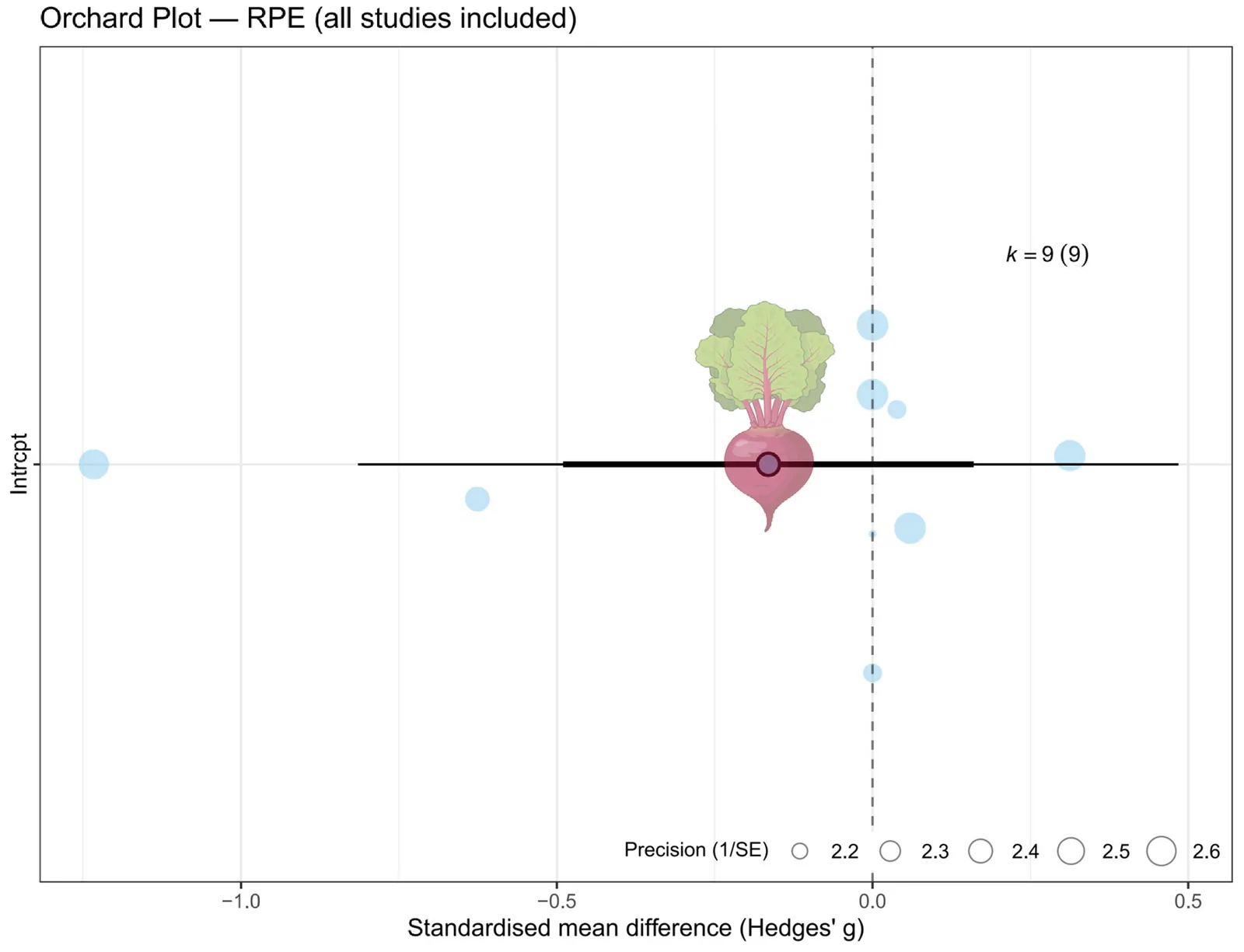
Orchard Plot of Rating of Perceived Exertion (Beetroot Juice vs. Placebo).

A total of 45 studies, spanning diverse sports including cycling, running, rowing, kayaking, swimming, cross-country skiing, triathlon, soccer, hockey, fencing, taekwondo, and CrossFit, contributed data to the meta-analysis across six outcome domains: PP, k = 12; MP, k = 17; VO_2_max, k = 26; HR, k = 19; LAC, k = 10; and RPE, k = 9. Random-effects meta-analyses revealed no statistically significant overall effect of dietary nitrate supplementation compared with placebo for any of the six exercise performance or physiological/perceptual response outcomes (Table 3). The pooled standardized mean differences (Hedges’ g) were small, and their 95% confidence intervals crossed zero: PP (g = 0.13, 95% CI −0.07 to 0.32, *p* = 0.20; τ^2^ = 0.0000, I^2^ = 0.00%, Q(11) = 12.90, *p* = 0.30) (Figure 3), MP (g = 0.04, 95% CI −0.13 to 0.21, *p* = 0.65; τ^2^ = 0.0000, I^2^ = 0.00%, Q(16) = 18.43, *p* = 0.30) (Figure 4), VO_2_max (g = −0.03, 95% CI −0.20 to 0.15, *p* = 0.77; τ^2^ = 0, I^2^ = 0.00%, Q(25) = 7.79, *p* = 0.9996) (Figure 5), HR (g = −0.08, 95% CI −0.27 to 0.10, *p* = 0.38; τ^2^ = 0, I^2^ = 0.00%, Q(18) = 4.88, *p* = 0.999) (Figure 6), LAC (g = 0.09, 95% CI −0.30 to 0.48, *p* = 0.64; τ^2^ = 0.2332, I^2^ = 59.79%, Q(9) = 22.65, *p* = 0.007) (Figure 7), and RPE (g = −0.17, 95% CI −0.49 to 0.16, *p* = 0.32; τ^2^ = 0.0823, I^2^ = 33.33%, Q(8) = 11.66, *p* = 0.167) (Figure 8). Heterogeneity was negligible for PP, MP, VO_2_max, and HR (I^2^ = 0.00%), moderate for LAC (I^2^ = 59.79%), and low-to-moderate for RPE (I^2^ = 33.33%).

#### 3.5.2. Subgroup Analyses

Subgroup analyses were performed for the categorical moderators supplement source, sex, sport type, exercise type, testing measure, and design type where sufficient data were available (≥3 studies per level). Most subgroup comparisons yielded non-significant interaction terms, indicating that the effect of nitrate supplementation did not differ significantly across levels of these moderators.

Supplement source. Subgroup analyses comparing beetroot juice with inorganic nitrate salts could be conducted for VO_2_max only. For VO_2_max, neither beetroot juice (g = −0.02, 95% CI −0.21 to 0.16) nor nitrate salts (g = −0.06, 95% CI −0.53 to 0.42) differed from placebo, and the interaction was not significant (*p* = 0.90). For PP, MP, HR, LAC, and RPE, there were insufficient studies in at least one supplement-source category to perform this subgroup analysis.

Sex. For PP, a significant positive effect was observed in male-only studies (g = 0.29, 95% CI 0.02 to 0.56, *p* = 0.04), but not in mixed-sex studies (g = −0.04, 95% CI −0.34 to 0.25, *p* = 0.78); however, the interaction test was not significant (*p* = 0.10). For VO_2_max, neither female (g = 0.09, 95% CI −0.37 to 0.54) nor male (g = −0.05, 95% CI −0.24 to 0.15) subgroups showed significant effects, and the interaction was not significant (*p* = 0.60). For HR, neither female (g = −0.19, 95% CI −0.65 to 0.27) nor male (g = −0.07, 95% CI −0.27 to 0.14) subgroups showed significant effects, and the interaction was not significant (*p* = 0.62).

Sport type. Only MP and VO_2_max had enough studies for sport subgroup analysis. For MP, the effect did not differ between cyclists (g = −0.04, 95% CI −0.49 to 0.40) and cyclists/triathletes (g = 0.12, 95% CI −0.27 to 0.50; interaction *p* = 0.60). For VO_2_max, effects were similar across cyclists (g = −0.21, 95% CI −0.69 to 0.28), cyclists/triathletes (g = −0.09, 95% CI −0.46 to 0.27), kayakers (g = −0.08, 95% CI −0.64 to 0.47), and runners (g = −0.04, 95% CI −0.48 to 0.41; interaction *p* = 0.96). No sport subgroup reached statistical significance.

Exercise type. When studies were grouped by exercise type based on established physiological frameworks, no significant interaction was observed for any outcome. For PP, effects were similar between endurance (g = 0.00, 95% CI −0.24 to 0.25) and strength/power (g = 0.25, 95% CI −0.18 to 0.68) categories (interaction *p* = 0.32). For MP, neither endurance (g = 0.01, 95% CI −0.19 to 0.21) nor strength/power (g = −0.05, 95% CI −0.51 to 0.40) categories showed significant effects (interaction *p* = 0.81). For HR, neither endurance (g = −0.13, 95% CI −0.34 to 0.09) nor intermittent team sports (g = 0.07, 95% CI −0.34 to 0.48) showed significant effects (interaction *p* = 0.41). For VO_2_max, the vast majority of studies involved endurance-type exercise (23 of 26), with insufficient studies in other categories to permit subgroup comparison. For LAC and RPE, insufficient numbers of studies per exercise type category prevented meaningful subgroup comparisons. A descriptive breakdown of individual study effects by sport and exercise type is provided in Supplementary Table S5, and a corresponding visualization of this breakdown is also provided in Supplementary Figure S2.

Testing measure. Subgroup analysis by testing measure was feasible only for VO_2_max (cycle ergometer vs. submaximal cycling trial). The comparison revealed no significant difference (cycle ergometer: g = 0.20, 95% CI −0.22 to 0.63; submaximal cycling trial: g = −0.31, 95% CI −0.80 to 0.18; interaction *p* = 0.12). For PP, MP, HR, LAC, and RPE, insufficient numbers of studies per testing measure prevented meaningful subgroup analysis.

Design type (crossover vs. parallel). Where sufficient data existed, we examined whether the effect differed between crossover and parallel designs. For VO_2_max, the effect was similar across designs (crossover: g = −0.05, 95% CI −0.23 to 0.14; parallel: g = 0.10; interaction *p* = 0.56). For HR, the point estimate was more negative in parallel studies (g = −0.57) than in crossover studies (g = −0.03), but the interaction was not significant (*p* = 0.11). Other outcomes had insufficient studies per design type for analysis.

#### 3.5.3. Sensitivity Analyses

Leave-one-out analyses demonstrated that the overall effect estimates were robust; removal of any single study did not materially change the direction or significance of the pooled effect for any outcome.

Sensitivity analyses examining the impact of the assumed pre–post correlation coefficient (r = 0.30, 0.50, 0.70) showed minimal changes in the pooled effects, confirming that the results are insensitive to this parameter. Furthermore, for crossover studies, we tested the sensitivity of the results to the assumed cross-condition correlation (r_cross = 0.30, 0.50, 0.70) between the change scores of the intervention and placebo conditions. The pooled effects remained consistent across all three assumptions for every outcome (Supplementary Figure S1), supporting the robustness of the independent-groups approximation used in the primary analysis.

#### 3.5.4. Publication Bias

Egger’s regression test and trim-and-fill analyses provide statistical evidence of funnel-plot asymmetry and potential small-study effects. However, these tests should not be interpreted as definitive proof of publication bias alone. In particular, Egger’s regression can be confounded by genuine between-study heterogeneity, especially when the number of studies is small (e.g., PP and MP); significant test results may therefore reflect true heterogeneity rather than publication bias and should be interpreted cautiously. For PP, Egger’s test was significant (z = 2.88, *p* = 0.02) and trim-and-fill imputed five missing studies, attenuating the pooled effect to essentially zero (g = −0.01, 95% CI −0.22 to 0.20); therefore, the publication-bias domain was downgraded to serious for PP. For MP, Egger’s test was also significant (z = 2.33, *p* = 0.03), indicating potential small-study effects, and the publication-bias domain was likewise downgraded to serious, even though trim-and-fill imputed no missing studies and the adjusted estimate remained unchanged. For the remaining outcomes, publication bias was rated as not serious because neither Egger’s test nor trim-and-fill indicated meaningful asymmetry or an influential impact on the pooled estimates.

## 4. Discussion

The present meta-analysis, encompassing 45 studies, found no statistically significant overall effect of dietary nitrate supplementation compared with placebo on any of the six exercise performance and physiological/perceptual response outcomes examined: peak power, mean power, maximal oxygen uptake, heart rate, blood lactate, and rating of perceived exertion. In exploratory subgroup analyses, a positive effect on peak power was observed in male-only studies, but the interaction between male-only and mixed-sex cohorts was not statistically significant. This finding should therefore be treated as hypothesis-generating rather than as evidence of a true sex-specific physiological effect. No other subgroup analyses reached statistical significance. Sensitivity analyses confirmed the robustness of the overall estimates, and trim-and-fill adjustments suggested that the apparent positive trend in peak power may be partially attributable to publication bias. Egger’s test also suggested small-study effects for mean power; however, trim-and-fill imputed no studies for this outcome, and the adjusted estimate was unchanged, suggesting that the small-study effect was less consequential for mean power. Importantly, the absence of statistically significant overall effects should not be equated with evidence of no effect. For several outcomes, the 95% confidence intervals were wide and included both the null and potentially meaningful small-to-moderate effects; the present findings should therefore be interpreted as imprecise null estimates.

Our finding that nitrate supplementation does not significantly improve exercise performance and physiological/perceptual response outcomes in athletes is consistent with several previous meta-analyses [3,4,93]. Specifically, prior work reported that nitrate supplementation was ergogenic in non-athletes (ES = 0.25, 95% CI 0.11 to 0.38) but not in athletes (ES = 0.04, 95% CI −0.05 to 0.15) [3]. Similarly, another study found a statistically significant relationship between training status and ergogenicity, with less-trained athletes (VO_2_max < 55 mL/kg/min) showing greater improvements in time trial performance after nitrate supplementation [4]. A further meta-analysis concluded that nitrate supplementation combined with exercise training did not enhance VO_2_peak or time to exhaustion beyond training alone [93]. These convergent findings suggest that the ergogenic potential of dietary nitrate is modulated by the athlete’s training status, with diminishing returns as aerobic fitness increases.

Several mechanistic explanations have been proposed for the attenuated response in well-trained athletes. One study demonstrated that the effects of nitrate supplementation on oxygen consumption and running performance are inversely related to individual aerobic fitness level, with no benefits observed in highly trained subjects (VO_2_max > 60 mL/kg/min) [94]. This may reflect higher baseline NO bioavailability in elite athletes, which blunts any additional benefit from exogenous nitrate [9]. Another commentary invoked the economic principle of diminishing returns, arguing that at the extreme performance levels of elite athletes, further input of any single component (including NO) yields progressively smaller improvements [95]. Furthermore, a previous hypothesis proposed that nitrate-derived NO production is not site-specific and may cause vasodilation of non-prioritized vascular beds, potentially diverting blood flow away from active muscles during maximal exertion when cardiac output is already limiting [10]. This mechanism could be particularly relevant in elite athletes who operate closer to their maximal cardiac output.

In addition, the role of the vascular endothelium and its interaction with NO deserves specific consideration in athletes with high-performance potential. The endothelium is the primary source of endogenous NO production via endothelial nitric oxide synthase, and shear-stress-induced endothelial responsiveness is a key determinant of exercise hyperaemia. In highly trained athletes, endothelial function is typically well adapted, which may further explain why exogenous nitrate supplementation produced no significant benefit in the present meta-analysis: if the endothelium is already operating near its functional ceiling, additional nitrate-derived NO is unlikely to meaningfully enhance vasodilation or metabolic efficiency. Conversely, athletes with transiently reduced endothelial function—for example, during periods of high training load or incomplete recovery—might be more responsive to supplementation. Future studies should therefore consider incorporating direct measures of endothelial function, such as flow-mediated dilation, to clarify how endothelial status modulates the ergogenic effects of dietary nitrate.

The absence of statistically significant pooled effects on PP and MP contrasts with some previous meta-analyses that reported small positive effects [18,19]. One such analysis found that nitrate supplementation improved MP output (standardized mean difference = 0.20, *p* = 0.02) and time to reach PP (standardized mean difference = 0.75, *p* = 0.02) during single and repeated bouts of high-intensity exercise [18]. Another reported improvements in repetitions to failure (standardized mean difference = 0.43, *p* = 0.002) and MP (standardized mean difference = 0.40, *p* = 0.004) during resistance exercise [19]. However, these analyses included a broader range of exercise modalities and often incorporated studies with less-trained participants. Our meta-analysis focused exclusively on athletes and included only studies that measured PP and MP during cycling or running tasks, which may explain the discrepancy. Additionally, the significant positive effect we observed for PP in males was attenuated after adjusting for publication bias, suggesting that small-study effects may have inflated the apparent benefit in this subgroup.

The lack of a statistically significant effect on VO_2_max aligns with previous findings [22], which noted contradictory data regarding the effects of nitrate supplementation on VO_2_max in athletes. While some individual studies have reported improvements in exercise economy and time to exhaustion at submaximal intensities, the translation to VO_2_max appears inconsistent. One review concluded that improvements in exercise tolerance could help with exhaustion over time, but the improvement in exercise economy was not clear [21]. Our results suggest that any potential benefits of nitrate supplementation on aerobic metabolism are not captured by changes in VO_2_max, which may be too coarse a measure to detect subtle improvements in mitochondrial efficiency or oxygen utilisation.

No statistically significant effects were observed for HR, LAC, or RPE. However, for HR (95% CI −0.27 to 0.10) and LAC (95% CI −0.30 to 0.48), the confidence intervals were relatively wide and included both null effects and small-to-moderate effects in either direction. These findings should therefore be interpreted as imprecise null estimates rather than as evidence that nitrate supplementation has no effect on these outcomes. For HR, the pooled effect was slightly more negative than in our preliminary analysis (g = −0.08 vs. −0.05), but remained non-significant. This contrasts with some individual studies that reported lower oxygen consumption during submaximal exercise after nitrate supplementation [59,96]. However, these effects appear to be more pronounced in untrained or recreationally active individuals. The non-significant findings for LAC are noteworthy given that a prior study reported greater LAC accumulation during intermittent sprint exercise after nitrate supplementation, which they interpreted as evidence of enhanced glycolytic flux [23]. Our meta-analysis, which included a heterogeneous mix of exercise protocols, did not replicate this finding, possibly because the lactate response is highly dependent on the specific exercise paradigm employed. The moderate heterogeneity observed for LAC (I^2^ = 59.79%) further underscores the variability across studies.

Our subgroup analysis showed a positive effect of nitrate supplementation on PP in male-only studies (g = 0.29, 95% CI 0.02 to 0.56, *p* = 0.04), but not in mixed-sex studies. However, the interaction test was not significant (*p* = 0.11), indicating that the difference between subgroups cannot be statistically distinguished from chance. Given the small number of studies, the absence of female-only cohorts, the exploratory nature of subgroup analyses, and the attenuation of the effect after trim-and-fill correction, this finding should not be interpreted as evidence of a sex-specific ergogenic effect. The limited evidence in female athletes remains an important gap [97], but definitive conclusions about sex differences require adequately powered studies specifically designed to test sex-by-supplement interactions [98]. Future research should therefore prioritise direct comparisons of male and female athletes under identical protocols.

The sport type and testing measure subgroup analyses did not reveal significant differences, suggesting that the absence of statistically significant effects is consistent across cycling, running, and kayaking modalities. Subgroup analysis by testing measure was feasible only for VO_2_max (cycle ergometer vs. submaximal cycling trial), and no significant difference was found (interaction *p* = 0.12). For LAC, insufficient studies per testing measure prevented subgroup analysis. This is somewhat surprising given that some commentators have argued that nitrate supplementation may be more beneficial for upper-body-dominant exercise or for sports involving repeated sprints [13,99]. Although the exercise type subgroup interactions were not significant, the descriptive distribution showed a positive point estimate for strength/power-type exercise in PP (g = 0.25), whereas endurance-type exercise was near zero. This may relate to muscle fibre type and nitrate effects on fast-twitch fibres, but the small number of studies precludes definitive conclusions. The lack of subgroup differences may reflect insufficient statistical power due to the small number of studies within each category, or it may indicate that the effect of nitrate supplementation is genuinely similar across exercise modalities in athletes.

We additionally compared beetroot juice with inorganic nitrate salts as a supplement-source subgroup analysis. This comparison was feasible only for VO_2_max. For VO_2_max, neither beetroot juice (g = −0.02, 95% CI −0.21 to 0.16) nor nitrate salts (g = −0.06, 95% CI −0.53 to 0.42) differed from placebo, and the interaction was not significant (*p* = 0.90). For PP, MP, HR, LAC, and RPE, insufficient studies in one or both source categories prevented a meaningful subgroup comparison. One included study used fresh spinach homogenate as the nitrate source; because this was the only non-beetroot, non-salt whole-food source, it was retained in the overall analyses but not included in the source-subgroup comparison. These results suggest that the delivery vehicle of dietary nitrate did not significantly modify the ergogenic response in athletes, although statistical power for this comparison was limited by the small number of inorganic nitrate salt studies.

To examine whether study design influenced our results, we conducted a subgroup analysis comparing crossover and parallel trials where sufficient data were available. For VO_2_max, the effect did not differ between designs (interaction *p* = 0.56). For HR, the point estimate was more negative in parallel studies (g = −0.57) than in crossover studies (g = −0.03), but the interaction was not significant (*p* = 0.11). These results suggest that the inclusion of both designs did not systematically bias our findings, and the independent-groups approximation used for crossover trials (which yields conservative variance estimates) did not materially affect the conclusions.

Several factors may explain why our results differ from meta-analyses that reported positive effects in non-athletes. First, the training status of participants is a critical moderator. A previous meta-analysis reported that the ergogenic effect of nitrate was largely confined to non-athletes, particularly during long-duration open-ended tests [3]. Second, the dose and duration of supplementation may be important. One recent umbrella review reported that acute supplementation (2–3 h pre-exercise) had a small effect (standardized mean difference = 0.20), while chronic supplementation (≥3 days) produced a negligible effect (standardized mean difference = 0.13) [100]. Our meta-analysis included both acute and chronic protocols, which may have diluted any acute benefits. Third, individual variability in the ability to reduce nitrate to NO_2_^−^, as previously highlighted [11], could mask group-level effects. The presence of “responders” and “non-responders” has been documented in several studies [5,101], and future research should adopt n-of-1 designs to identify individuals who genuinely benefit. Additionally, the conservative independent-groups approximation used for the 41 crossover trials may have reduced statistical power and contributed to the null findings, as discussed in the Limitations Section.

The potential for nitrate supplementation to improve performance in specific contexts deserves further investigation. Some researchers argued that athletes competing in events held at altitude, particularly those with frequent transitions in work rate, may benefit from nitrate supplementation due to enhanced blood flow and microvascular PO_2_ in fast-twitch muscles [12]. Another commentary suggested that nitrate might mitigate hypoxic pulmonary vasoconstriction and improve cerebral blood flow, although the evidence in trained athletes remains inconclusive [102]. A further proposal suggested that nitrate could improve thermoregulation through vasodilation and sweat regulation, which might benefit athletes training in hot environments [103]. These hypotheses warrant rigorous testing in well-designed randomised controlled trials.

Future studies should address several methodological shortcomings identified in this meta-analysis. First, prospective registration of study protocols and pre-specification of primary outcomes are essential to reduce the risk of selective reporting. Second, studies should include adequate sample sizes based on a priori power calculations to detect the small ESs that are realistic for nitrate supplementation. Third, researchers should standardise the measurement of plasma nitrate and NO_2_ concentrations to better characterise the pharmacokinetic response and identify responders. Fourth, the inclusion of female athletes and older athletes (master athletes) should be prioritised, as these populations may respond differently. Fifth, studies should investigate the dose–response relationship in elite athletes, as higher doses (8.3–16.4 mmol) may be required to elevate plasma NO_2_^−^ sufficiently [100]. Sixth, future meta-analyses should aim to obtain individual participant data from crossover trials to enable proper paired-effect-size analyses, although our sensitivity analyses suggest that the independent-groups approximation used here yields robust conclusions. Finally, the interaction between nitrate supplementation and other ergogenic aids, particularly caffeine, warrants further exploration, as some evidence suggests that caffeine may attenuate the effects of nitrate [22]. Taken together, while the current evidence does not demonstrate an ergogenic effect of dietary nitrate in athletes, it also does not exclude small benefits in specific contexts or subgroups. Future research should aim to reduce uncertainty through larger, well-controlled trials with precise outcome measures.

### Limitations

This meta-analysis has several limitations that should be acknowledged. First, the overall risk of bias across included studies was predominantly rated as “some concerns” per RoB 2 criteria [36], with only 1 study rated as low risk and 44 as some concerns. The main sources of bias were insufficient reporting of random sequence generation (only 9 of 45 studies adequately described methods) and lack of prospective registration (only 5 studies were pre-registered). Consequently, the certainty of evidence was graded as “moderate” for VO_2_max and HR, “low” for PP and MP, and “very low” for LAC and RPE following GRADE guidance [104]. Second, moderate heterogeneity was observed for LAC (I^2^ = 59.79%) and low-to-moderate heterogeneity for RPE (I^2^ = 33.33%); while I^2^ values < 30% are conventionally considered low [43], residual heterogeneity may stem from unmeasured moderators such as training background or supplementation timing that were not uniformly reported across studies. We also note that the I^2^ values of 0.00% for PP, MP, VO_2_max, and HR reflect REML estimates of τ^2^ truncated at zero, indicating no detectable between-study variance given the power of the Q-test; small amounts of true heterogeneity below the detection threshold cannot be excluded. The heterogeneity observed for LAC and RPE, the outcomes with the fewest studies, is physiologically plausible and may also reflect unstable variance estimates at small k. In addition, sport type subgroup analyses were only feasible for MP and VO_2_max; for the other four outcomes, too-few studies per sport category prevented formal testing. Sport-specific differences in energy-system demands and muscle recruitment therefore remain a plausible but unresolved source of heterogeneity, particularly given the context-specific ergogenic potential of nitrate. Third, the number of studies available for subgroup analyses was limited, particularly for sex, sport type, and testing measure comparisons, which reduced statistical power to detect interaction effects. Subgroup meta-analytic tests have inherently lower power than primary pooled analyses when cell sizes are small [105], which may explain why the male-specific PP effect did not yield a significant interaction despite the within-group estimate being positive. Similarly, the supplement-source subgroup comparison could only be performed for VO_2_max, and included only one study using other nitrate-rich vegetables, precluding a robust test of whether the nitrate source modulates the effect. Fourth, publication bias was detected for PP and MP via Egger’s regression test [49], and although trim-and-fill adjustments did not materially alter the conclusions for most outcomes, the possibility of unpublished null results—particularly from small trials with non-significant findings—cannot be excluded [50]. For PP, trim-and-fill imputed five missing studies and reduced the pooled effect to essentially zero (g = −0.01, 95% CI −0.22 to 0.20); for MP, no studies were imputed and the estimate remained unchanged. For VO_2_max, HR, LAC, and RPE, trim-and-fill imputed 5, 0, 2, and 2 studies, respectively, but the adjusted estimates remained non-significant. This aligns with documented overrepresentation of positive small trials in the sports nutrition literature [28]. Fifth, the meta-analysis relied on aggregate data rather than individual participant data (IPD), which precluded more nuanced analyses of moderators such as baseline fitness (e.g., VO_2_max strata), nitrate dose per kilogram of body mass, and plasma NO_2_ response. IPD approaches are better suited to quantify individual-level modifiers of treatment response [106], which is particularly relevant for nitrate supplementation given the established roles of oral nitrate reductase activity and baseline NO bioavailability in determining ergogenicity [11,94]. Sixth, 41 of the 45 included studies used crossover designs, and the independent-groups approximation applied to these trials represents a conservative analytical choice that systematically inflates the variance of effect estimates and reduces statistical power relative to a proper paired analysis. Although this approach was selected transparently because within-participant correlations were rarely reported, it likely attenuated our ability to detect small but real effects, and may therefore partly explain the null overall findings for several outcomes. Access to IPD would have permitted paired-effect meta-analysis and more precise estimates, but IPD were not available for the present study. Seventh, the inclusion of studies with varying supplementation protocols (acute vs. chronic, nitrate doses ranging from ~6 to ~16 mmol) and exercise modalities (laboratory ergometry, on-water testing, intermittent vs. continuous efforts) may have introduced unmeasured confounding that could not be fully controlled. Dose–response relationships for nitrate are not yet firmly established in athletes [100], and ESs may differ across testing contexts in ways our aggregate-data approach could not fully disaggregate [2]. In addition, the small number of inorganic nitrate salts studies limited the precision of the supplement-source subgroup estimates. Seventh, we acknowledge that the outcomes examined in this meta-analysis—PP, MP, VO_2_max, HR, LAC, and RPE—span distinct physiological constructs, ranging from predominantly anaerobic power (PP, MP) to maximal cardiorespiratory capacity (VO_2_max) and acute physiological/perceptual responses (HR, LAC, RPE). We deliberately did not classify them under a single “aerobic capacity” umbrella, as such a framework would be physiologically imprecise. Instead, we report them as complementary indices that together characterise the broad effects of nitrate supplementation on exercise performance and the physiological/perceptual responses to standardized exercise testing in athletes.

## 5. Conclusions

In conclusion, this meta-analysis of 45 randomized controlled trials found no statistically significant pooled effect of dietary nitrate supplementation on exercise performance or physiological/perceptual responses in athletes, including PP, MP, VO_2_max, HR, LAC, and RPE. The supplement-source subgroup analysis indicated no significant difference between beetroot juice and inorganic nitrate salts for VO_2_max (the only outcome for which this comparison was feasible). The positive effect on PP in male-only studies was not statistically robust (interaction *p* = 0.11; attenuated after trim-and-fill adjustment) and should be interpreted as exploratory. These findings are consistent with previous meta-analyses suggesting that the ergogenic benefits of dietary nitrate may be largely confined to non-athletes or less-trained individuals. However, given that several confidence intervals included potentially meaningful effects and the certainty of evidence ranged from moderate to very low across outcomes, our results should not be interpreted as definitive evidence of no effect. Future research should focus on identifying individual characteristics that predict responsiveness, optimising dosing strategies, and exploring specific exercise contexts (e.g., altitude, intermittent sprint protocols) where nitrate supplementation may confer a meaningful advantage.

## Figures and Tables

**Table 1:** Eligibility criteria.

	Inclusion Criteria	Exclusion Criteria
Participant	Athletes classified as Tier 2 (regional/provincial level) or higher per the participant calibration framework [26], from any sport, sex, or age group.	Non-athletes (e.g., recreationally active, sedentary participants not meeting Tier 2 threshold). Athletes with a history of musculoskeletal injury in the past 6 months that could affect exercise performance or physiological measurement [27].
Intervention	Acute (single-dose) or chronic (≥2 days repeated dosing) oral supplementation with nitrate-rich beetroot juice, dietary nitrate (e.g., sodium nitrate, potassium nitrate), or nitrate salts. For the purposes of this review, “dietary nitrate supplementation” encompasses all oral nitrate sources, including nitrate-rich beetroot juice and inorganic nitrate salts.	Co-supplementation with other ergogenic aids (e.g., caffeine, beta-alanine, creatine) unless the nitrate-only condition could be isolated from the combined intervention. Intravenous, topical, or other non-oral nitrate administration routes.
Comparator	Placebo condition matched for appearance, taste, and packaging (e.g., nitrate-depleted beetroot juice, blackcurrant juice, inert cellulose capsules).	No placebo control (e.g., water-only, open-label design). Active comparator only (e.g., caffeine, other supplements with no placebo arm) [28].
Outcome	Pre- and post-supplementation measurement of at least one of the following exercise performance or physiological/perceptual response indices: peak power (PP), mean power (MP), maximal oxygen uptake (VO_2_max), heart rate (HR), blood lactate (LAC), or rating of perceived exertion (RPE). These outcomes represent commonly used measures of exercise performance (PP, MP) and acute physiological (VO_2_max, HR, LAC) or perceptual (RPE) responses to exercise, rather than being limited to strictly aerobic capacity markers. They were selected to capture a broad spectrum of responses to standardized exercise testing in athletes. Exercise tests must involve sustained aerobic or high-intensity intermittent effort with single-bout duration >30 s (e.g., 30-s Wingate test, graded exercise test to exhaustion, submaximal constant-load cycling/running, Yo-Yo IR1, time-trial protocols) [2,29].	Outcomes unrelated to exercise performance and physiological/perceptual responses (e.g., maximal isometric/isokinetic strength, <10-s sprint, cognitive function, resting blood pressure). Missing pre/post means, standard deviations, or sample sizes that precluded effect size calculation after contacting authors.
Study design	Randomized, placebo-controlled, crossover or parallel-group trials.	Non-randomized designs (quasi-experimental, observational, case series). Grey literature (theses, conference abstracts, unpublished datasets) to ensure peer-reviewed, methodologically vetted evidence and consistent reporting quality [24].

**Table 2:** Certainty of evidence assessment.

Certainty Assessment						No. of Participants			Certainty
No. of studies	Risk of bias	Inconsistency	Indirectness	Imprecision	Publication bias	SP	PLA	Absolute (95% CI)	
SP vs. PLA (PP)									
12	Serious	Not serious	Not serious	Not serious	Serious	212	212	ES 0.13 (−0.07 to 0.32)	Low
SP vs. PLA (MP)									
17	Serious	Not serious	Not serious	Not serious	Serious	265	265	ES 0.04 (−0.13 to 0.21)	Low
SP vs. PLA (VO_2_max)									
26	Serious	Not serious	Not serious	Not serious	Not serious	262	262	ES −0.03 (−0.20 to 0.15)	Moderate
SP vs. PLA (HR)									
19	Serious	Not serious	Not serious	Not serious	Not serious	229	229	ES −0.08 (−0.27 to 0.10)	Moderate
SP vs. PLA (LAC)									
10	Serious	Serious	Not serious	Serious	Not serious	134	134	ES 0.09 (−0.30 to 0.48)	Very Low
SP vs. PLA (RPE)									
9	Serious	Serious	Not serious	Serious	Not serious	113	113	ES −0.17 (−0.49 to 0.16)	Very Low

SP: supplement group; PLA: placebo group; CI: confidence interval; ES: effect size; PP: peak power; MP: mean power; VO_2_max: maximal oxygen uptake; HR: heart rate; LAC: blood lactate; RPE: rating of perceived exertion. For crossover trials, the SP and PLA columns represent the same participants under each condition; the number of participants for GRADE imprecision refers to unique individuals, and the two columns are not summated.

**Table 3:** Overall effect estimates for each exercise performance and physiological/perceptual response outcome.

Outcome	k	Hedges’ g	95% CI	*p*-Value	I^2^ (%)	τ^2^
PP	12	0.127	−0.065, 0.319	0.196	0	0
MP	17	0.040	−0.132, 0.212	0.647	0	0
VO_2_max	26	−0.026	−0.198, 0.146	0.769	0	0
HR	19	−0.082	−0.266, 0.101	0.380	0	0
LAC	10	0.093	−0.296, 0.482	0.641	60	0.233
RPE	9	−0.165	−0.490, 0.160	0.320	33	0.082

I^2^ was computed from the REML estimate of τ^2^. Zero I^2^ values indicate no detectable between-study variance given the available power, rather than proof of exact homogeneity.

## Data Availability

The complete dataset generated and analyzed during the current study is available in the Supplementary Information and at the Open Science Framework repository (https://osf.io/8stk7/overview (accessed on 30 June 2026)).

## Supplementary Materials

The following supporting information can be downloaded at: https://www.mdpi.com/article/10.3390/sports14100466/s1. References in supplementary files: Balsalobre et al., 2018 [51]; Bescós et al., 2012 [52]; Bescós et al., 2011 [53]; Cermak et al., 2012a [54]; Cermak et al., 2012b [55]; Christensen et al., 2013 [6]; Manuel V. Garnacho-Castaño et al., 2022 [56]; Hoon et al., 2014 [57]; Lane et al., 2014 [58]; Lansley et al., 2011 [59]; McQuillan et al., 2017a [60]; McQuillan et al., 2017b [61]; Nybäck et al., 2017 [62]; Pawlak-Chaouch et al., 2019 [63]; Peacock et al., 2012 [64]; Rokkedal et al., 2019 [65]; Shannon et al., 2017 [66]; Vahid Giv et al., 2022 [67]; Stacey P.A. Forbes et al., 2021 [68]; Colin R. Carriker et al., 2016 [69]; Zafer Kavcı et al., 2024 [70]; Aitor Viribay et al., 2024 [71]; Manuel V. Garnacho-Castaño et al., 2024 [72]; Jekaterina Neteca et al., 2024 [73]; Johannes Pollex et al., 2025 [74]; Scott A. Conger et al., 2021 [75]; de Castro et al., 2019 [76]; Peeling et al., 2015 [77]; Muggeridge et al., 2013 [78]; Muggeridge et al., 2014 [79]; Arnold, J et al., 2015 [7]; Boorsma et al., 2014 [5]; Handzlik, L et al., 2013 [80]; Glaister, M et al., 2015 [81]; Esen et al., 2023 [82]; Oskarsson et al., 2018 [83]; Burgos et al., 2021 [84]; Berjisian et al., 2022 [85]; Dumar et al., 2021 [86]; Jodra et al., 2020 [87]; Jonvik et al., 2018 [88]; Kramer et al., 2022 [89]; Reynolds et al., 2020 [90]; Rimer et al., 2017 [91]; Melike Nur Eroglu et al., 2026 [92].
