# Supplementary material for: Dietary Nitrate Supplementation and Exercise Performance in Athletes: A Systematic Review and Meta-Analysis of Randomised Controlled Trials
Source: Sports (Basel). 2026 Oct 20;14(10):466. doi: 10.3390/sports14100466 (PMC13709359; doi:10.3390/sports14100466)
Supplement: Supplementary file 1 [file sports-14-00466-s001.zip › Supplementary Figure S1.pdf]

Forest Plot — PP (k = 12)

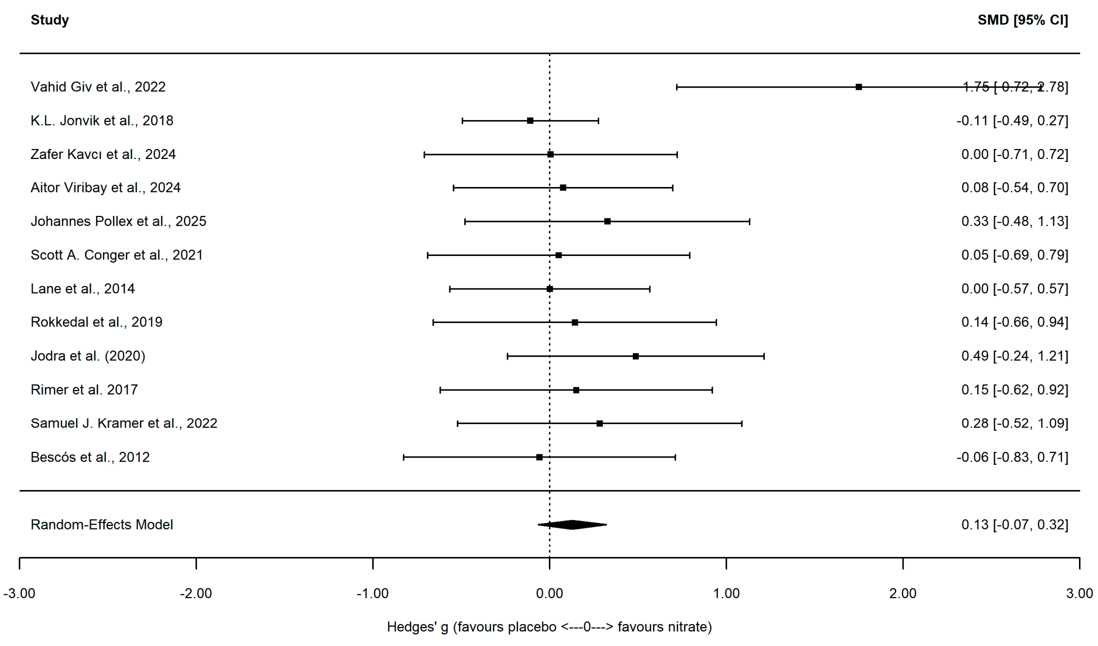

Forest Plot — MP (k = 17)

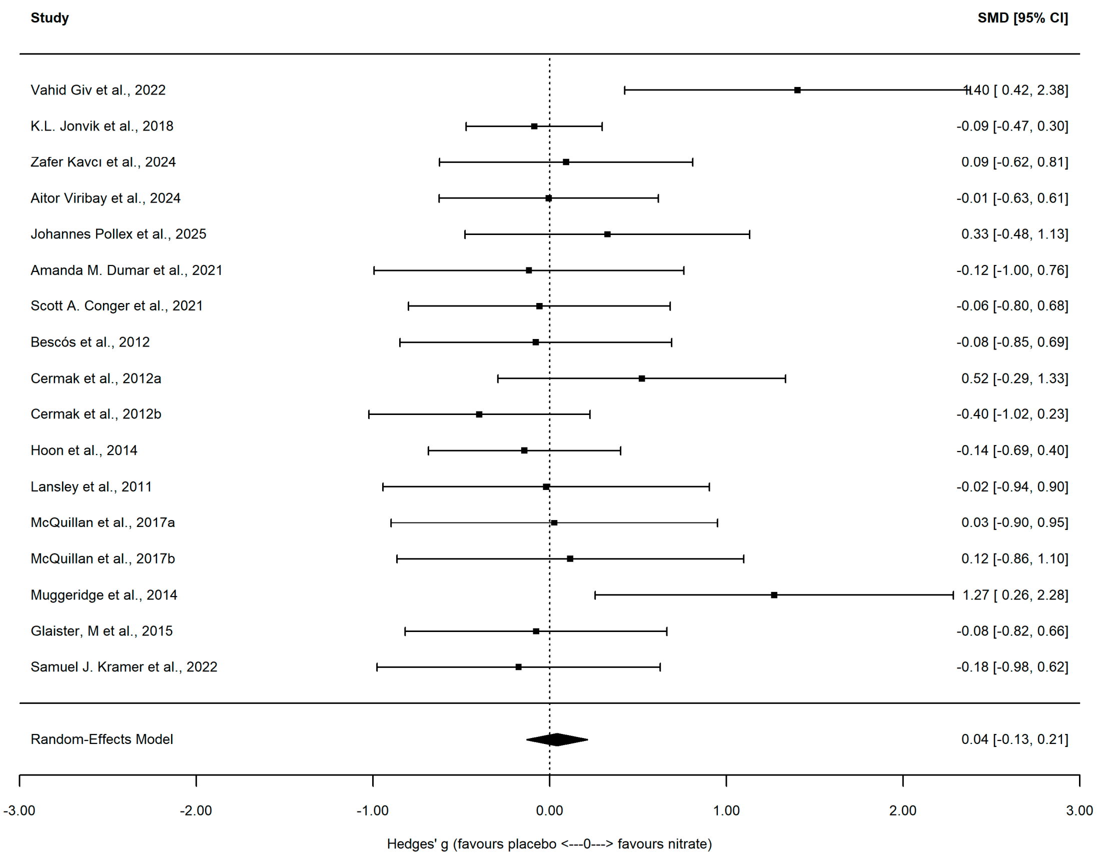

Forest Plot — VO2 (k = 26)

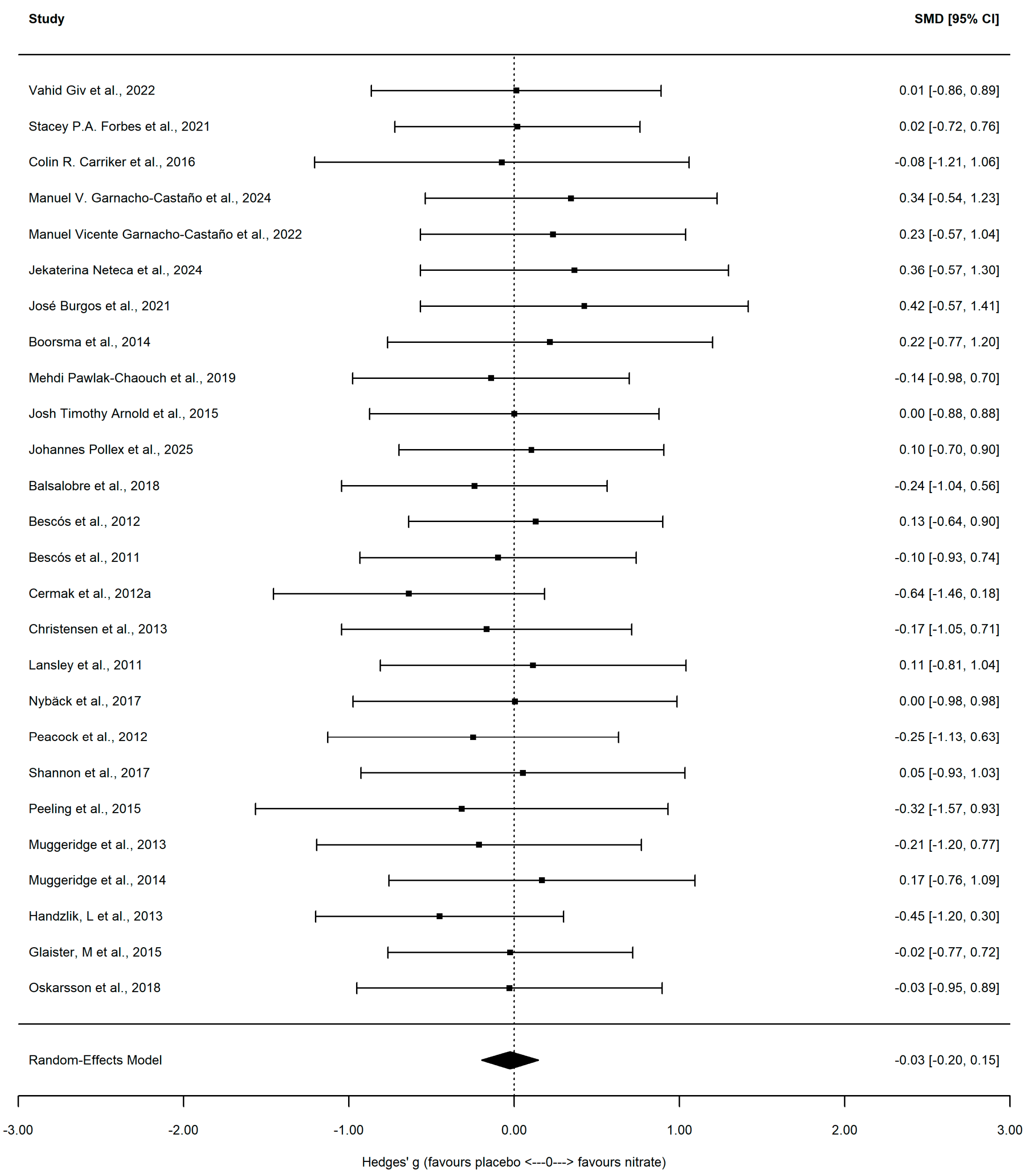

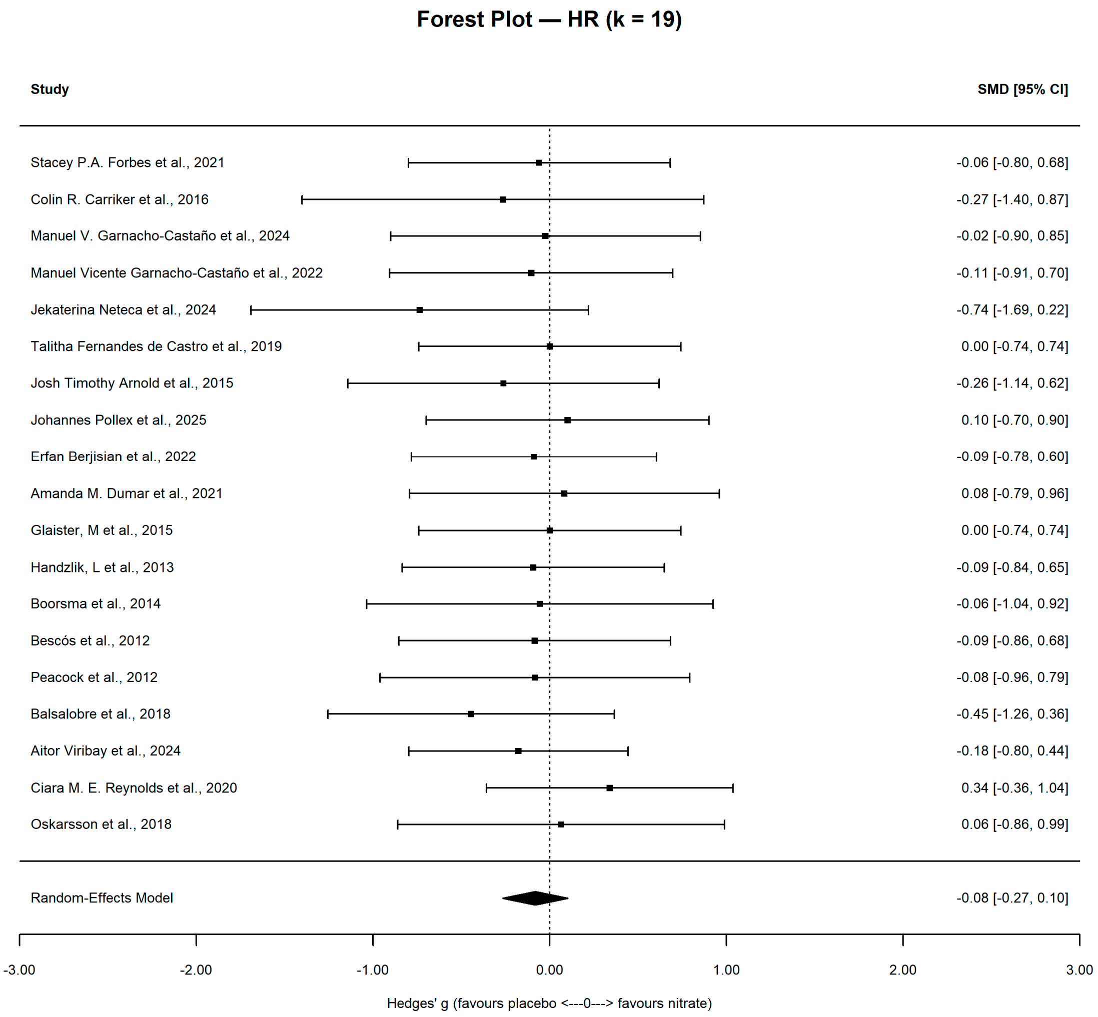
Forest Plot — HR (k = 19)
Study
SMD [95% CI]
Stacey P.A. Forbes et al., 2021 -0.06 [-0.80, 0.68]
Colin R. Carriker et al., 2016 -0.27 [-1.40, 0.87]
Manuel V. Garnacho-Castaño et al., 2024 -0.02 [-0.90, 0.85]
Manuel Vicente Garnacho-Castaño et al., 2022 -0.11 [-0.91, 0.70]
Jekaterina Neteca et al., 2024 -0.74 [-1.69, 0.22]
Talitha Fernandes de Castro et al., 2019 0.00 [-0.74, 0.74]
Josh Timothy Arnold et al., 2015 -0.26 [-1.14, 0.62]
Johannes Pollex et al., 2025 0.10 [-0.70, 0.90]
Erfan Berjisian et al., 2022 -0.09 [-0.78, 0.60]
Amanda M. Dumar et al., 2021 0.08 [-0.79, 0.96]
Glaister, M et al., 2015 0.00 [-0.74, 0.74]
Handzlik, L et al., 2013 -0.09 [-0.84, 0.65]
Boorsma et al., 2014 -0.06 [-1.04, 0.92]
Bescós et al., 2012 -0.09 [-0.86, 0.68]
Peacock et al., 2012 -0.08 [-0.96, 0.79]
Balsalobre et al., 2018 -0.45 [-1.26, 0.36]
Aitor Viribay et al., 2024 -0.18 [-0.80, 0.44]
Ciara M. E. Reynolds et al., 2020 0.34 [-0.36, 1.04]
Oskarsson et al., 2018 0.06 [-0.86, 0.99]
Random-Effects Model -0.08 [-0.27, 0.10]
-3.00
-2.00
-1.00
0.00
1.00
2.00
3.00
Hedges' g (favours placebo <---0---> favours nitrate)

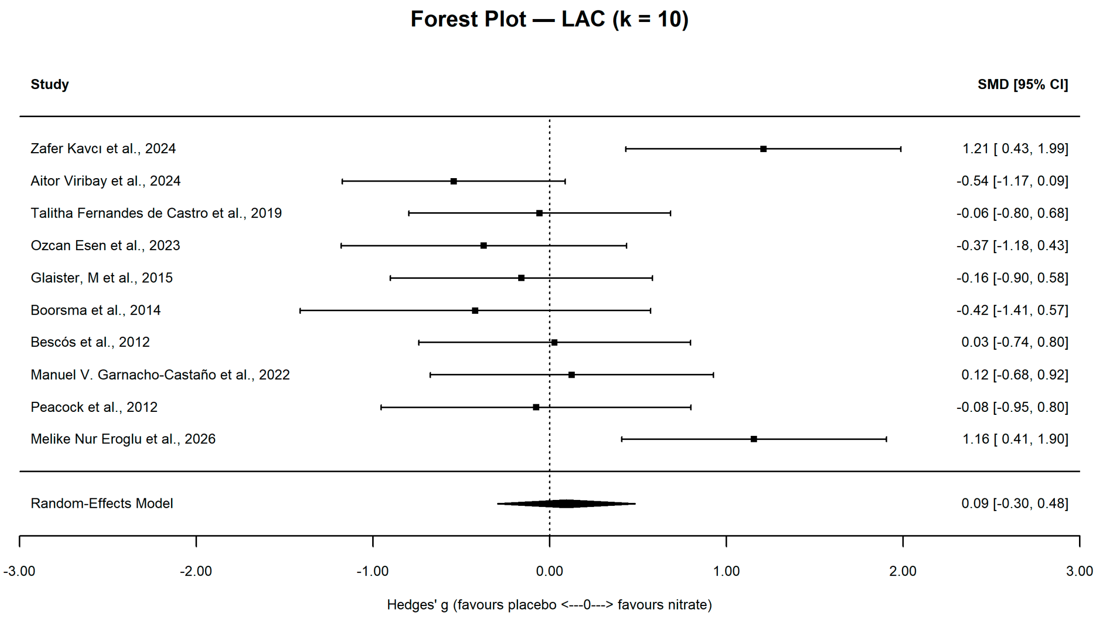
Forest Plot — LAC (k = 10)
Study
SMD [95% CI]
Zafer Kavcı et al., 2024 1.21 [0.43, 1.99]
Aitor Viribay et al., 2024 -0.54 [-1.17, 0.09]
Talitha Fernandes de Castro et al., 2019 -0.06 [-0.80, 0.68]
Ozcan Esen et al., 2023 -0.37 [-1.18, 0.43]
Glaister, M et al., 2015 -0.16 [-0.90, 0.58]
Boorsma et al., 2014 -0.42 [-1.41, 0.57]
Bescós et al., 2012 0.03 [-0.74, 0.80]
Manuel V. Garnacho-Castaño et al., 2022 0.12 [-0.68, 0.92]
Peacock et al., 2012 -0.08 [-0.95, 0.80]
Melike Nur Eroglu et al., 2026 1.16 [0.41, 1.90]
Random-Effects Model 0.09 [-0.30, 0.48]
-3.00
-2.00
-1.00
0.00
1.00
2.00
3.00
Hedges' g (favours placebo <---0---> favours nitrate)

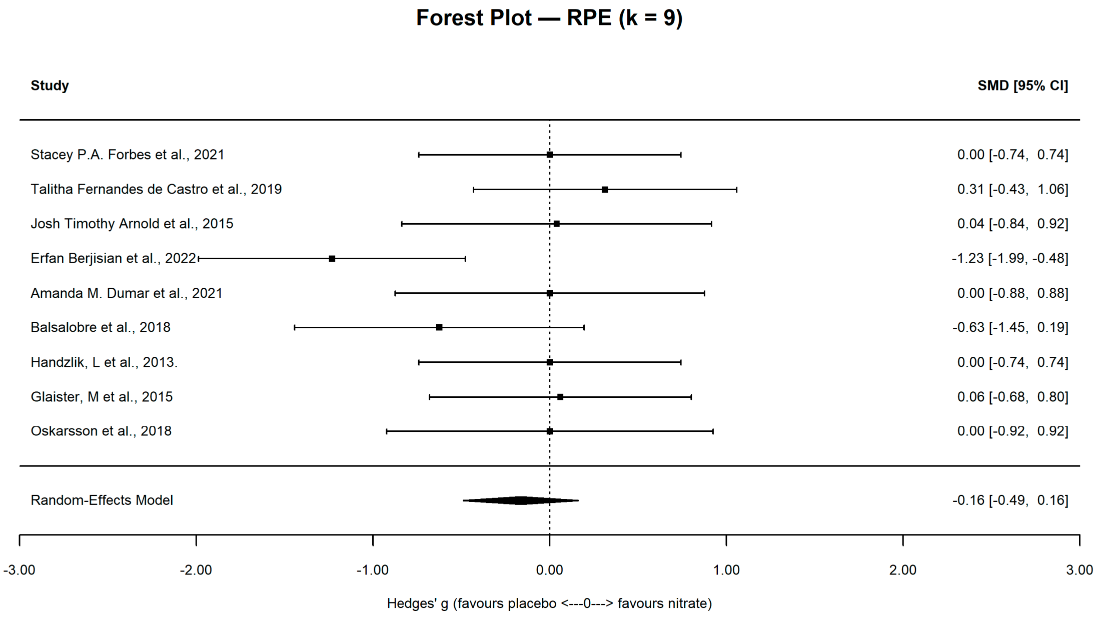
Forest Plot — RPE (k = 9)
Study
SMD [95% CI]
Stacey P.A. Forbes et al., 2021
0.00 [-0.74, 0.74]
Talitha Fernandes de Castro et al., 2019
0.31 [-0.43, 1.06]
Josh Timothy Arnold et al., 2015
0.04 [-0.84, 0.92]
Erfan Berjisian et al., 2022
-1.23 [-1.99, -0.48]
Amanda M. Dumar et al., 2021
0.00 [-0.88, 0.88]
Balsalobre et al., 2018
-0.63 [-1.45, 0.19]
Handzlik, L et al., 2013.
0.00 [-0.74, 0.74]
Glaister, M et al., 2015
0.06 [-0.68, 0.80]
Oskarsson et al., 2018
0.00 [-0.92, 0.92]
Random-Effects Model
-0.16 [-0.49, 0.16]
-3.00
-2.00
-1.00
0.00
1.00
2.00
3.00
Hedges' g (favours placebo <---0---> favours nitrate)
