# Supplementary material for: Dietary Nitrate Supplementation and Exercise Performance in Athletes: A Systematic Review and Meta-Analysis of Randomised Controlled Trials
Source: Sports (Basel). 2026 Oct 20;14(10):466. doi: 10.3390/sports14100466 (PMC13709359; doi:10.3390/sports14100466)
Supplement: Supplementary file 1 [file sports-14-00466-s001.zip › Supplementary Figure S2.pdf]

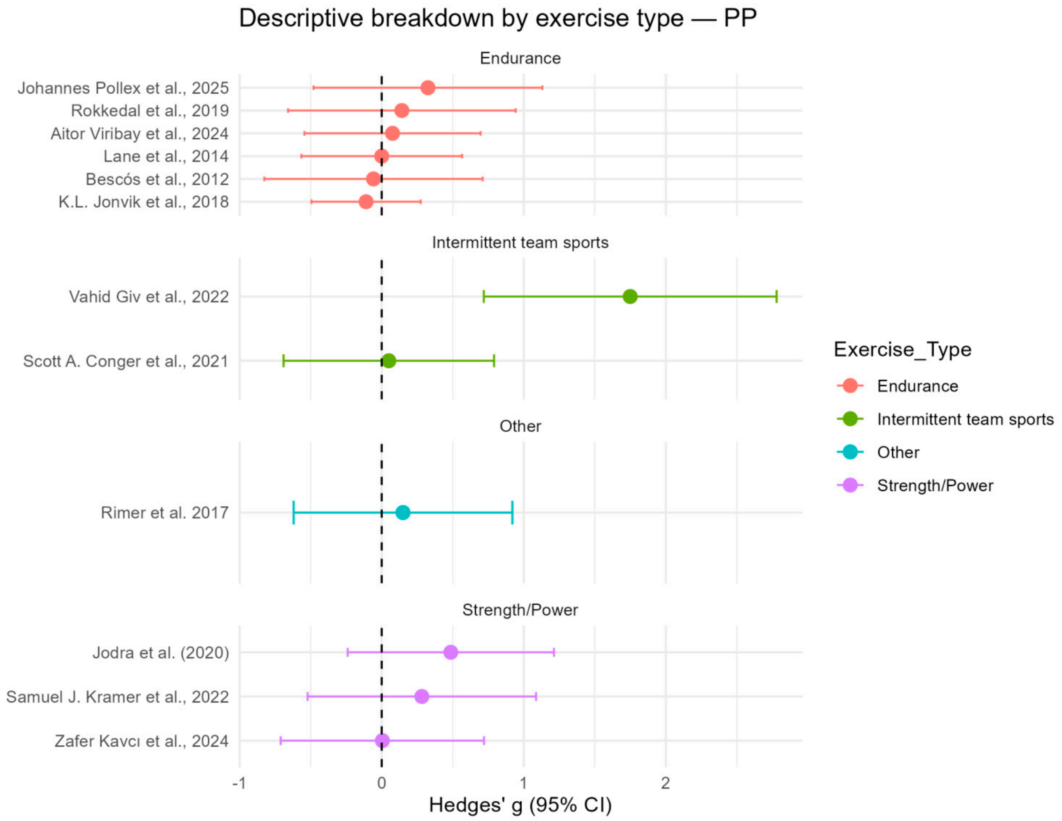
Descriptive breakdown by exercise type — PP
Endurance
Johannes Pollex et al., 2025
Rokkedal et al., 2019
Aitor Viribay et al., 2024
Lane et al., 2014
Bescós et al., 2012
K.L. Jonvik et al., 2018
Intermittent team sports
Vahid Giv et al., 2022
Scott A. Conger et al., 2021
Other
Rimer et al. 2017
Strength/Power
Jodra et al. (2020)
Samuel J. Kramer et al., 2022
Zafer Kavcı et al., 2024
-1
0
1
2
Hedges' g (95% CI)
Exercise_Type
Endurance
Intermittent team sports
Other
Strength/Power

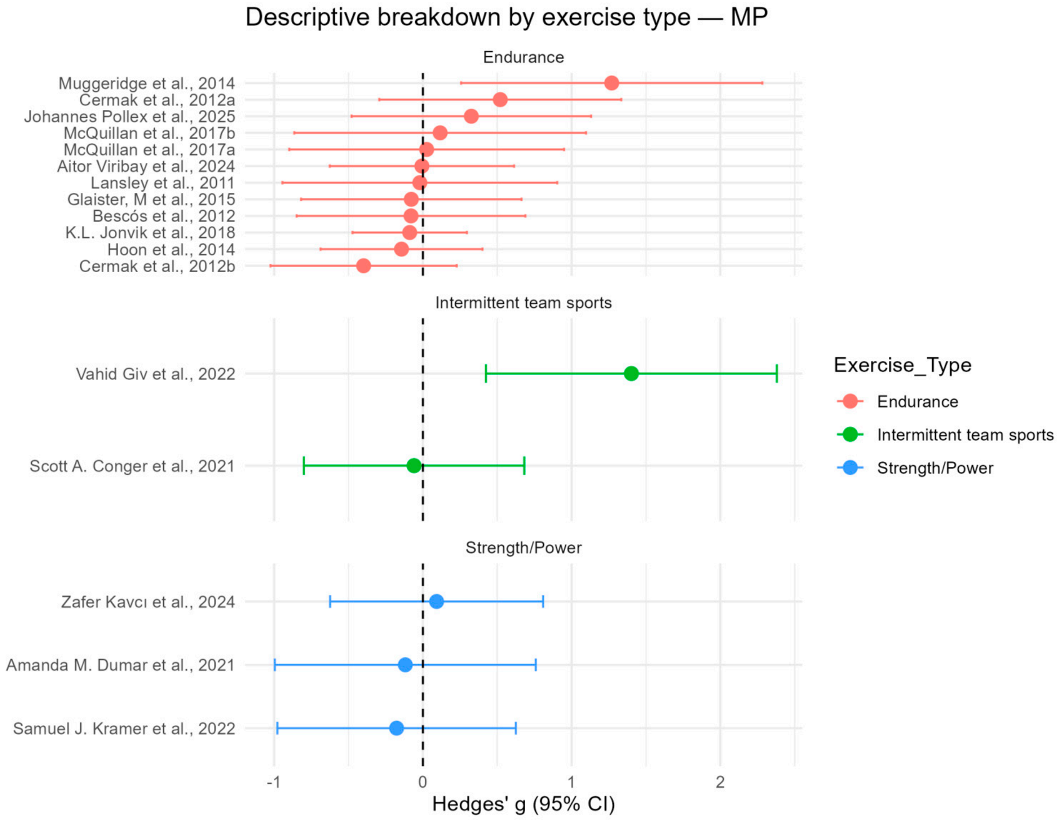
Descriptive breakdown by exercise type — MP
Endurance
Muggeridge et al., 2014
Cermak et al., 2012a
Johannes Pollex et al., 2025
McQuillan et al., 2017b
McQuillan et al., 2017a
Aitor Viribay et al., 2024
Lansley et al., 2011
Glaister, M et al., 2015
Bescós et al., 2012
K.L. Jonvik et al., 2018
Hoon et al., 2014
Cermak et al., 2012b
Intermittent team sports
Vahid Giv et al., 2022
Scott A. Conger et al., 2021
Strength/Power
Zafer Kavcı et al., 2024
Amanda M. Dumar et al., 2021
Samuel J. Kramer et al., 2022
-1
0
1
2
Hedges' g (95% CI)
Exercise_Type
Endurance
Intermittent team sports
Strength/Power

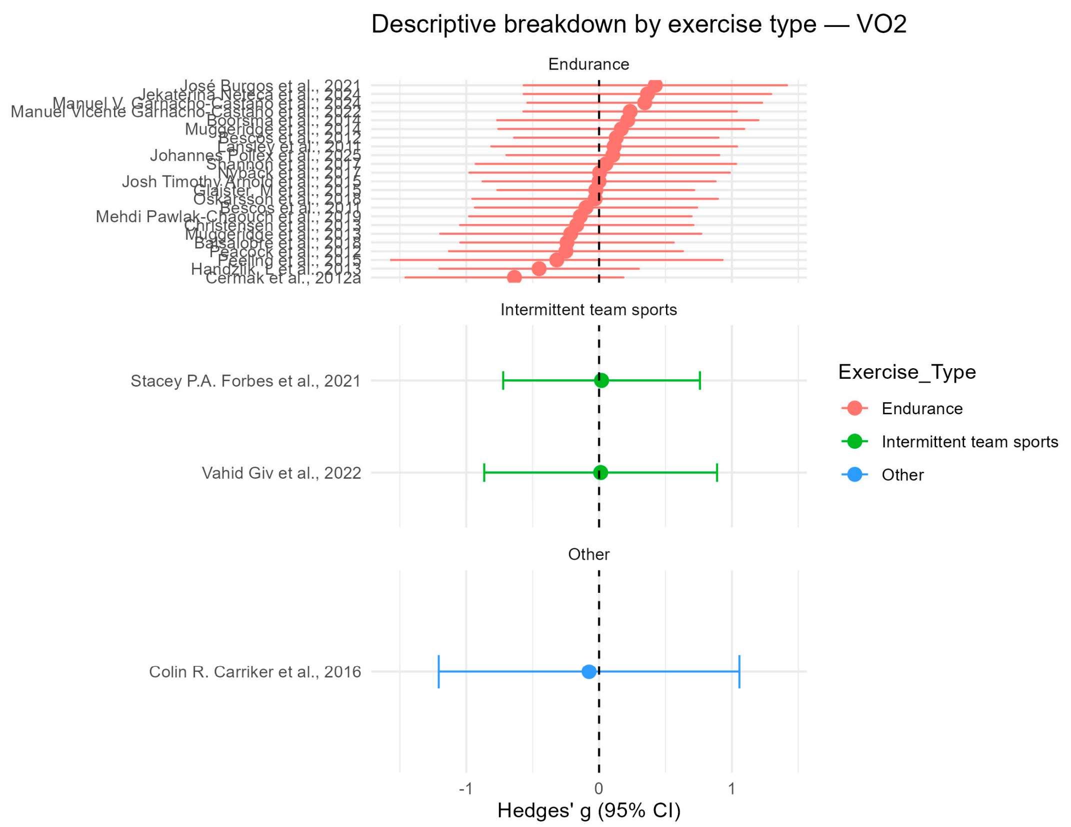

Descriptive breakdown by exercise type — VO2
Endurance
José Burgos et al., 2021
Jekaterina Neteca et al., 2024
Manuel V. Garnacho-Castaño et al., 2024
Manuel Vicente Garnacho-Castaño et al., 2022
Boorsma et al., 2014
Muggeridge et al., 2014
Bescos et al., 2012
Lansley et al., 2011
Johannes Pollex et al., 2025
Shannon et al., 2017
Nyback et al., 2017
Josh Timothy Arnold et al., 2015
Glaister, M et al., 2015
Oskarsson et al., 2018
Bescos et al., 2011
Mehdi Pawlak-Chaouch et al., 2019
Christensen et al., 2013
Muggeridge et al., 2013
Balsalobre et al., 2018
Peacock et al., 2012
Peeling et al., 2015
Handzlik, L et al., 2013
Cermak et al., 2012a
Intermittent team sports
Stacey P.A. Forbes et al., 2021
Vahid Giv et al., 2022
Other
Colin R. Carriker et al., 2016
-1
0
1
Hedges' g (95% CI)
Exercise_Type
Endurance
Intermittent team sports
Other

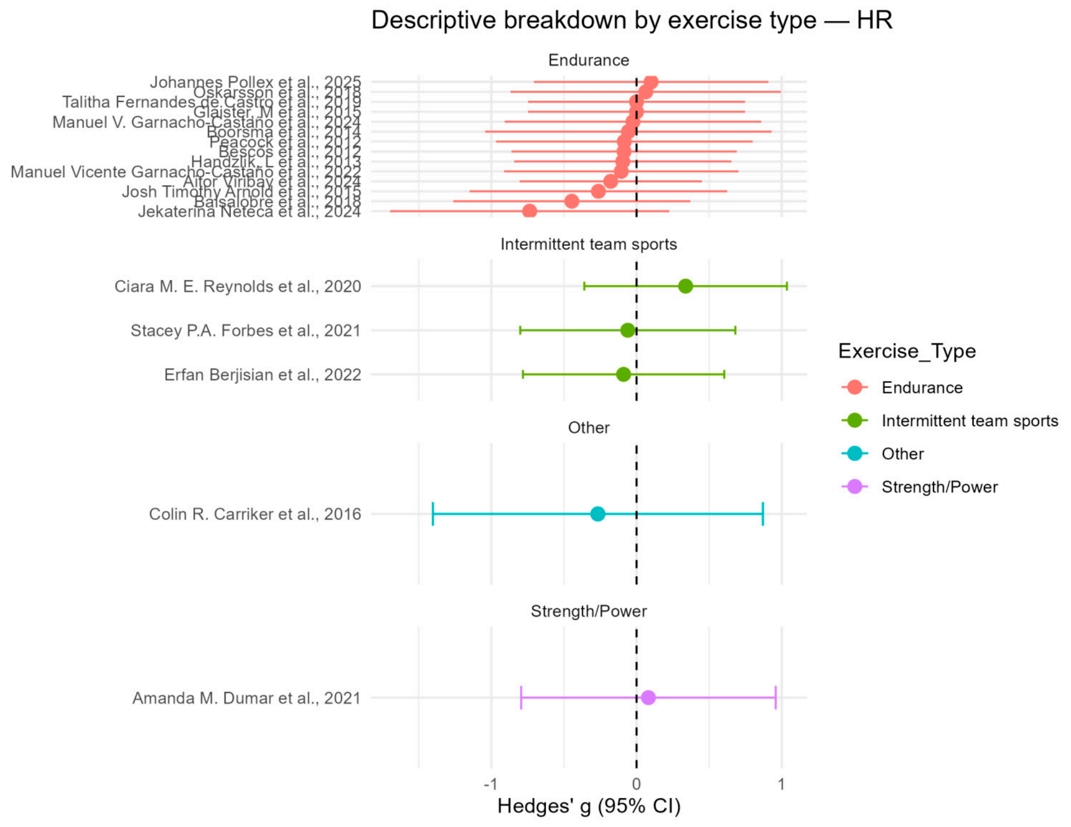

Descriptive breakdown by exercise type — HR
Endurance
Johannes Pollex et al., 2025
Oskarsson et al., 2018
Talitha Fernandes de Castro et al., 2019
Glaister, M et al., 2015
Manuel V. Garnacho-Castaño et al., 2024
Boorsma et al., 2014
Peacock et al., 2012
Bescos et al., 2012
Handzlik, L et al., 2013
Manuel Vicente Garnacho-Castaño et al., 2022
Aitor Viribay et al., 2024
Josh Timothy Arnold et al., 2015
Balsalobre et al., 2018
Jekaterina Neteca et al., 2024
Intermittent team sports
Ciara M. E. Reynolds et al., 2020
Stacey P.A. Forbes et al., 2021
Erfan Berjisian et al., 2022
Other
Colin R. Carriker et al., 2016
Strength/Power
Amanda M. Dumar et al., 2021
-1
0
1
Hedges' g (95% CI)
Exercise_Type
Endurance
Intermittent team sports
Other
Strength/Power

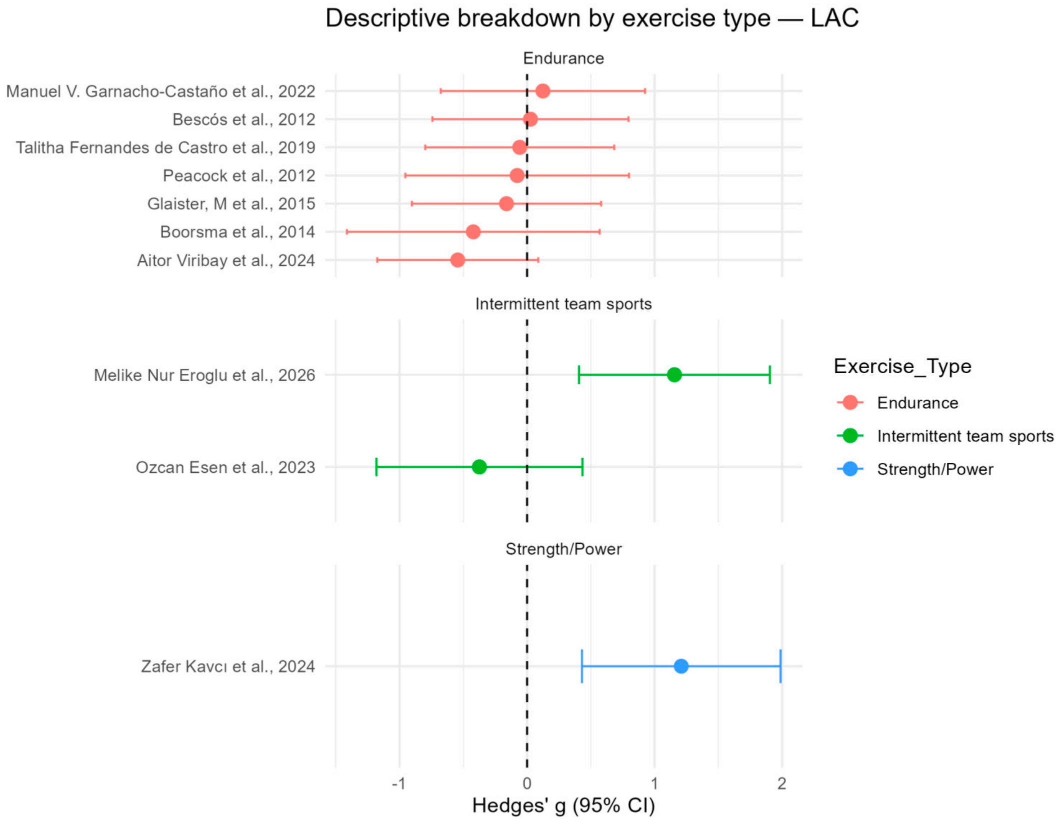
Descriptive breakdown by exercise type — LAC
Endurance
Manuel V. Garnacho-Castaño et al., 2022
Bescós et al., 2012
Talitha Fernandes de Castro et al., 2019
Peacock et al., 2012
Glaister, M et al., 2015
Boorsma et al., 2014
Aitor Viribay et al., 2024
Intermittent team sports
Melike Nur Eroglu et al., 2026
Ozcan Esen et al., 2023
Strength/Power
Zafer Kavcı et al., 2024
-1
0
1
2
Hedges' g (95% CI)
Exercise_Type
Endurance
Intermittent team sports
Strength/Power

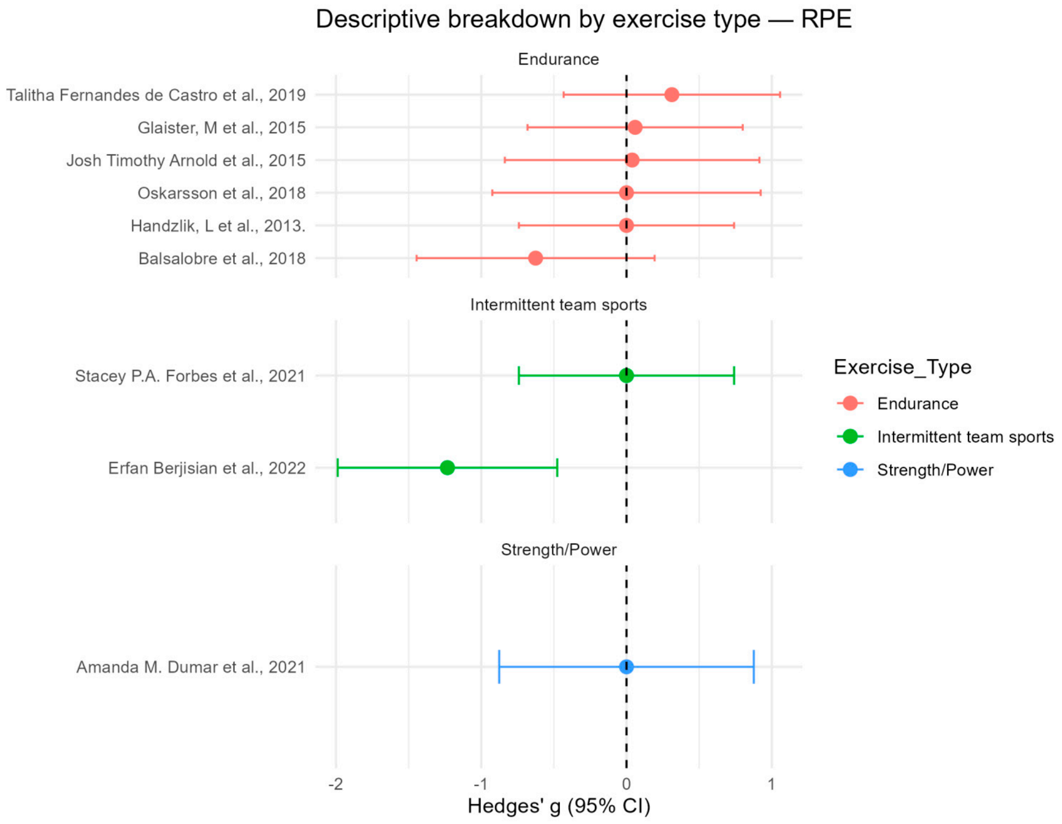
Descriptive breakdown by exercise type — RPE
Endurance
Talitha Fernandes de Castro et al., 2019
Glaister, M et al., 2015
Josh Timothy Arnold et al., 2015
Oskarsson et al., 2018
Handzlik, L et al., 2013.
Balsalobre et al., 2018
Intermittent team sports
Stacey P.A. Forbes et al., 2021
Erfan Berjisian et al., 2022
Strength/Power
Amanda M. Dumar et al., 2021
-2
-1
0
1
Hedges' g (95% CI)
Exercise_Type
Endurance
Intermittent team sports
Strength/Power
