# Supplementary material for: Dietary Nitrate Supplementation and Exercise Performance in Athletes: A Systematic Review and Meta-Analysis of Randomised Controlled Trials
Source: Sports (Basel). 2026 Oct 20;14(10):466. doi: 10.3390/sports14100466 (PMC13709359; doi:10.3390/sports14100466)
Supplement: Supplementary file 1 [file sports-14-00466-s001.zip › Supplementary Table S1.pdf]

Supplementary Table S1. Sensitivity analysis of the assumed cross-condition correlation (r_cross) in crossover studies.

Outcome	r_cross	Hedges' g	95% CI
PP	0.3	0.068	–0.067, 0.202
	0.5	0.078	–0.057, 0.214
	0.7	0.113	–0.035, 0.262
MP	0.3	–0.011	–0.135, 0.114
	0.5	–0.017	–0.142, 0.108
	0.7	–0.014	–0.158, 0.130
VO_2max	0.3	–0.039	–0.171, 0.094
	0.5	–0.044	–0.177, 0.089
	0.7	–0.052	–0.186, 0.082
HR	0.3	–0.031	–0.168, 0.105
	0.5	–0.037	–0.174, 0.100
	0.7	–0.048	–0.185, 0.089
LAC	0.3	–0.039	–0.312, 0.235
	0.5	–0.045	–0.359, 0.268
	0.7	–0.057	–0.443, 0.330
RPE	0.3	–0.072	–0.331, 0.187
	0.5	–0.075	–0.361, 0.211
	0.7	–0.079	–0.408, 0.250

Note: The pooled effect was re-estimated using adjusted variances that accounted for the assumed cross-condition correlation between change scores of the intervention and placebo conditions. The primary analysis used an independent-groups approximation (equivalent to r_cross = 0). All estimates remained non-significant and consistent across the three assumptions, supporting the robustness of the primary results.
