# Supplementary material for: Dietary Nitrate Supplementation and Exercise Performance in Athletes: A Systematic Review and Meta-Analysis of Randomised Controlled Trials
Source: Sports (Basel). 2026 Oct 20;14(10):466. doi: 10.3390/sports14100466 (PMC13709359; doi:10.3390/sports14100466)
Supplement: Supplementary file 1 [file sports-14-00466-s001.zip › Supplementary Table S2.pdf]

PubMed

("beetroot juice" OR beetroot OR "red beet" OR "beta vulgaris" OR "dietary nitrate" OR "nitrate supplementation" OR "sodium nitrate" OR "potassium nitrate" OR "nitric oxide" OR nitrate$)

AND

(exercise OR performance OR "physical performance" OR "sport$" OR athlete$ OR "peak power" OR "mean power" OR "wingate" OR "vo2max" OR "maximal oxygen uptake" OR "heart rate" OR "blood lactate" OR "lactate" OR RPE OR "perceived exertion" OR "aerobic" OR "anaerobic" OR sprint$ OR time trial$)

AND

(random$ OR RCT OR "controlled trial" OR crossover OR "placebo")

Scopus

TITLE-ABS-KEY((beetroot OR "beetroot juice" OR "red beet" OR "beta vulgaris" OR "dietary nitrate" OR nitrate OR "nitric oxide")

AND (exercise OR performance OR sport OR athlete OR "peak power" OR "mean power" OR wingate OR vo2max OR "oxygen uptake" OR "heart rate" OR lactate OR RPE OR "perceived exertion" OR sprint OR "time trial")

AND (random* OR "controlled trial" OR crossover OR placebo))

SPORTDiscus

TX ("beetroot juice" OR beetroot OR "red beet" OR "beta vulgaris" OR "dietary nitrate" OR nitrate OR "nitric oxide")

AND TX (exercise OR performance OR sport OR athlete OR "peak power" OR "mean power" OR wingate OR vo2max OR "maximal oxygen uptake" OR "heart rate" OR "blood lactate" OR lactate OR RPE OR "perceived exertion" OR sprint OR "time trial")

AND TX (random* OR "controlled trial" OR crossover OR placebo)

Web of Science

TOPIC: ((beetroot OR "beetroot juice" OR "red beet" OR "beta vulgaris" OR "dietary nitrate" OR nitrate OR "nitric oxide")

AND (exercise OR performance OR sport OR athlete OR "peak power" OR "mean power" OR wingate OR vo2max OR "oxygen uptake" OR "heart rate" OR lactate OR RPE OR "perceived exertion" OR sprint OR "time trial")

AND (random* OR "controlled trial" OR crossover OR placebo))
