# Supplementary material for: Dietary Nitrate Supplementation and Exercise Performance in Athletes: A Systematic Review and Meta-Analysis of Randomised Controlled Trials
Source: Sports (Basel). 2026 Oct 20;14(10):466. doi: 10.3390/sports14100466 (PMC13709359; doi:10.3390/sports14100466)
Supplement: Supplementary file 1 [file sports-14-00466-s001.zip › Supplementary Table S4.pdf]

Supplementary Table S4. Excluded Articles after Full Text Read.

Full-text articles evaluated for eligibility (n = 332). Exclude records (n = 298).

First author (year)	Article title	Exclusion reason
**Study design or study content is not in compliance (n = 129)**		
Garnacho-Castaño	Garnacho-Castaño, M., Sánchez-Nuño, S., Molina-Raya, L., Carbonell, T., Maté-Muñoz,	Study design or study content is not
Garnacho-Castaño	Garnacho-Castaño, M. V., Palau-Salvà, G., Serra-Payá, N., Ruiz-Hermosel, M., Berbell,	Study design or study content is not
Griffiths (2023)	Griffiths, A., Alhulaefi, S., Hayes, E., Matu, J., Brandt, K., Watson, A., Siervo, M., &	Study design or study content is not
Gruska (2024)	Gruska, N., Sarmento, H., Martinho, D., Field, A., & Massart, A. (2024). Enhancing	Study design or study content is not
Guler (2026)	Guler, I., Sajedi, H., Fidel, B., Kaya, E., Akpinar, S., Khodadadi, D., Laher, I., & Babaei,	Study design or study content is not
Hagele (2026)	Hagele, A. M., Sunderland, K. L., Mumford, P. W., & Kerksick, C. M. (2026). Effects of	Study design or study content is not
He (2026)	He, Y., Ge, X., Tang, C., Wang, Y., Huang, H., Leng, B., & Zhang, C. (2026). Dietary	Study design or study content is not
Hemmati (2024)	Hemmati, H., Alkasasbeh, W., Hemmatinafar, M., Salesi, M., Pirmohammadi, S.,	Study design or study content is not
Hogwood (2023)	Hogwood, A. C., Anderson, K. C., Ortiz de Zevallos, J., Paterson, C., Weltman, A., &	Study design or study content is not
Hogwood (2024)	Hogwood, A. C., Ortiz De Zevallos, J., Kruse, K., Buckley, M., De Guzman, J., DeJong	Study design or study content is not
Jaafar (2016)	Jaafar, M., Maojie, J., Trina, T., & Tracy, T. (2016). The Effects of Beetroot Juice	Study design or study content is not
Jędrejko (2024)	Jędrejko, M., Jędrejko, K., Gómez-Renaud, V. M., Kała, K., & Muszyńska, B. (2024).	Study design or study content is not
Jo (2019)	Jo, E., Fischer, M., Auslander, A., Beigarten, A., Daggy, B., Hansen, K., Kessler, L.,	Study design or study content is not
Jones (2012)	Jones, A. M., Bailey, S. J., & Vanhatalo, A. (2012). Dietary nitrate and O_2 consumption	Study design or study content is not
Jones (2021)	Jones, A. M., Vanhatalo, A., Seals, D. R., Rossman, M. J., Piknova, B., & Jonvik, K. L.	Study design or study content is not
Jones (2022)	Jones, L., Bailey, S. J., Rowland, S. N., Alsharif, N., Shannon, O. M., & Clifford, T.	Study design or study content is not
Jonvik (2021)	Jonvik, K., Hoogervorst, D., Peelen, H., De Niet, M., Verdijk, L., Van Loon, L., & van	Study design or study content is not
Jonvik (2017)	Jonvik, K., Nyakayiru, J., van Dijk, J., Wardenaar, F., van Loon, L., & Verdijk, L.	Study design or study content is not
Jonvik (2015)	Jonvik, K., Nyakayiru, J., van Loon, L., & Verdijk, L. (2015). Can elite athletes benefit	Study design or study content is not
Jurado-Castro	Jurado-Castro, J., Campos-Perez, J., Ranchal-Sanchez, A., Durán-López, N., &	Study design or study content is not
Kaviani (2020)	Kaviani, M., & Koozehchian, M. (2020). The Effects Of Beetroot And Tart Cherry	Study design or study content is not
Kelly (2013)	Kelly, J., Vanhatalo, A., Wilkerson, D. P., Wylie, L. J., & Jones, A. M. (2013). Effects of	Study design or study content is not
Kenjale (2011)	Kenjale, A. A., Ham, K. L., Stabler, T., Robbins, J. L., Johnson, J. L., Vanbruggen, M.,	Study design or study content is not
Kent (2019)	Kent, G., Dawson, B., McNaughton, L., Cox, G., Burke, L., & Peeling, P. (2019). The	Study design or study content is not
Kent (2018)	Kent, G. L., Dawson, B., Cox, G. R., Abbiss, C. R., Smith, K. J., Croft, K. D., Lim, Z. X.,	Study design or study content is not
Kent (2018)	Kent, G. L., Dawson, B., Cox, G. R., Burke, L. M., Eastwood, A., Croft, K. D., &	Study design or study content is not
Khosravi (2021)	Khosravi, S., Ahmadizad, S., Yekaninejad, M., Karami, M., & Djafarian, K. (2021). The	Study design or study content is not
Kirk (2017)	Kirk, B., Mitchell, J., Jackson, M., Amirabdollahian, F., Alizadehkhaiyat, O., & Clifford,	Study design or study content is not
Kuennen (2015)	Kuennen, M., Jansen, L., Gillum, T., Granados, J., Castillo, W., Nabiyar, A., &	Study design or study content is not
Lago-Rodríguez	Lago-Rodríguez, Á., Domínguez, R., Ramos-Álvarez, J. J., Tobal, F. M., Jodra, P., Tan,	Study design or study content is not
Lansley (2011)	Lansley, K. E., Winyard, P. G., Fulford, J., Vanhatalo, A., Bailey, S. J., Blackwell, J. R.,	Study design or study content is not
Larsen (2019)	Larsen, R. G., Thomsen, J. M., Hirata, R. P., Steffensen, R., Poulsen, E. R., Frøkjaer, J.	Study design or study content is not
Lee (2015)	Lee, J.-S., Stebbins, C. L., Jung, E., Nho, H., Kim, J.-K., Chang, M.-J., & Choi, H.-M.	Study design or study content is not
Lipert (2022)	Lipert, A., Szadkowska, I., Matusiak-Wieczorek, E., & Kochan, E. (2022). The Effect of	Study design or study content is not
Liu (2025)	Liu, X., Huo, L., Wang, F., Wang, T., Rong, W., & He, Y. (2025). Caffeine and Beetroot	Study design or study content is not
Liubertas (2020)	Liubertas, T., Kairaitis, R., Stasiule, L., Capkauskiene, S., Stasiulis, A., Viskelis, P.,	Study design or study content is not
López-Samanes	López-Samanes, A., Gómez Parra, A., Moreno-Pérez, V., & Courel-Ibáñez, J. (2020).	Study design or study content is not
López-Samanes	López-Samanes, A., Ramos-Alvarez, J., Miguel-Tobal, F., Gaos, S., Jodra, P., Arranz-	Study design or study content is not
Lowings (2017)	Lowings, S., Shannon, O. M., Deighton, K., Matu, J., & Barlow, M. J. (2017). Effect of	Study design or study content is not
MacLeod (2015)	MacLeod, K. E., Nugent, S. F., Barr, S. I., Koehle, M. S., Sporer, B. C., & MacInnis, M.	Study design or study content is not
Macuh (2023)	Macuh, M., Kojić, N., & Knap, B. (2023). The Effects of Nitrate Supplementation on	Study design or study content is not
Martin (2014)	Martin, K., Smee, D., Thompson, K., & Rattray, B. (2014). No Improvement of	Study design or study content is not
McDonagh (2015)	McDonagh, S. T. J., Wylie, L. J., Winyard, P. G., Vanhatalo, A., & Jones, A. M. (2015).	Study design or study content is not
McLellan (2026)	McLellan, A., Acton, J., O'Donnell, E., Rowland, S., Shepherd, A., Perissiou, M., James,	Study design or study content is not
Mcmahon (2023)	Mcmahon, N., Brooker, P., Pavey, T., & Leveritt, M. (2023). Assessment of dietary	Study design or study content is not
Meng (2025)	Meng, F., Liu, Y., Qiu, B., & Li, J. (2025). Does Nitrate Supplementation Improve	Study design or study content is not
Moreno-Heredero	Moreno-Heredero, B., Morencos, E., Morais, J., Barbosa, T., & Veiga, S. (2024). A	Study design or study content is not
Morishima (2022)	Morishima, T., Iemitsu, M., Fujie, S., & Ochi, E. (2022). Prior beetroot juice ingestion	Study design or study content is not
Morishima (2025)	Morishima, T., Mizuno, S., Murakami, G., Yamazaki, R., & Ochi, E. (2025). Prior	Study design or study content is not
Mosher (2020)	Mosher, S. L., Gough, L. A., Deb, S., Saunders, B., Mc Naughton, L. R., Brown, D. R.,	Study design or study content is not
Mrakic-Sposta	Mrakic-Sposta, S., Vezzoli, A., Parenza, M., Magno, M., D'Angelo, G., Nannipieri, F.,	Study design or study content is not
Muñoz (2025)	Muñoz, A., de la Rubia, A., Lorenzo-Calvo, J., Karayigit, R., Garcés-Rimón, M., López-	Study design or study content is not
Murphy (2022)	Murphy, J., Rushing, R., Sumner, J., & Hackney, C. (2022). Dietary Supplements for	Study design or study content is not
Murphy (2012)	Murphy, M., Eliot, K., Heuertz, R., & Weiss, E. (2012). Whole Beetroot Consumption	Study design or study content is not
Nie (2025)	Nie, R., Zhang, G., Kong, J., Wang, X., Xie, Z., Lin, L., Li, T., & Liang, Y. (2025).	Study design or study content is not
Nojoumi (2026)	Nojoumi, M., Jafari, A., Kakhki, A., Esfehani, A., Rafiei, H., Kerksick, C., Owoeye, O.,	Study design or study content is not
Nojoumi (2026)	Nojoumi, M., Kakhki, A. H., Sardar, M. A., Jafari, A., Esfehani, A. J., & Rezvani, R.	Study design or study content is not
Nyakayiru (2017)	Nyakayiru, J., Jonvik, K., Trommelen, J., Pinckaers, P., Senden, J., van Loon, L., &	Study design or study content is not
Nyakayiru (2020)	Nyakayiru, J., van Loon, L. J. C., & Verdijk, L. B. (2020). Could intramuscular storage	Study design or study content is not
Ortiz de Zevallos	Ortiz de Zevallos, J., Hogwood, A. C., Kruse, K., De Guzman, J., Buckley, M., DeJong	Study design or study content is not
Oue (2025)	Oue, A., Ito, M., Iimura, Y., Serizawa, N., Miyakoshi, Y., & Ota, M. (2025). Effect of	Study design or study content is not
Papadopoulos	Papadopoulos, S., Dipla, K., Triantafyllou, A., Nikolaidis, M. G., Kyparos, A.,	Study design or study content is not
Peeling (2019)	Peeling, P., Castell, L., Derave, W., de Hon, O., & Burke, L. (2019). Sports Foods and	Study design or study content is not

Pinna (2014)	Pinna, M., Roberto, S., Milia, R., Marongiu, E., Olla, S., Loi, A., Migliaccio, G., Padulo,	Study design or study content is not
Poortmans (2015)	Poortmans, J., Gualano, B., & Carpentier, A. (2015). Nitrate supplementation and human	Study design or study content is not
Porcelli (2016)	Porcelli, S., Pugliese, L., Rejc, E., Pavei, G., Bonato, M., Montorsi, M., La Torre, A.,	Study design or study content is not
Porcelli (2015)	Porcelli, S., Ramaglia, M., Bellistri, G., Pavei, G., Pugliese, L., Montorsi, M., Rasica, L.,	Study design or study content is not
Puype (2015)	Puype, J., Ramaekers, M., Van Thienen, R., Deldicque, L., & Hespel, P. (2015). No	Study design or study content is not
Qin (2026)	Qin, G., & Kim, S. (2026). Effects of beetroot juice supplementation on maximal oxygen	Study design or study content is not
Ranchal-Sanchez	Ranchal-Sanchez, A., Diaz-Bernier, V., de la Florida-Villagran, C., Llorente-Cantarero,	Study design or study content is not
Rasica (2025)	Rasica, L., Colosio, M., Ferri, A., Baldassarre, G., Easton, C., Burleigh, M., Vernillo, G.,	Study design or study content is not
Reynolds (2016)	Reynolds, C., Halpenny, C., Hughes, C., Jordan, S., Quinn, A., & Egan, B. (2016). Acute	Study design or study content is not
Richard (2018)	Richard, P., Koziris, L., Charbonneau, M., Naulleau, C., Tremblay, J., & Billaut, F.	Study design or study content is not
Robinson (2021)	Robinson, G., Killer, S., Stoyanov, Z., Stephens, H., Read, L., James, L., & Bailey, S.	Study design or study content is not
Rodríguez-	Rodríguez-Fernández, A., Castillo, D., Raya-González, J., Domínguez, R., & Bailey, S.	Study design or study content is not
Rojas-Valverde	Rojas-Valverde, D., Montoya-Rodríguez, J., Azofeifa-Mora, C., & Sanchez-Urena, B.	Study design or study content is not
Rokkedal-Lausch	Rokkedal-Lausch, T., Franch, J., Poulsen, M., Thomsen, L., Weitzberg, E., Kamavuako,	Study design or study content is not
Rothschild (2020)	Rothschild, J. A., & Bishop, D. J. (2020). Effects of Dietary Supplements on Adaptations	Study design or study content is not
Rowland (2022)	Rowland, S. N., Da Boit, M., Tan, R., Robinson, G. P., O'Donnell, E., James, L. J., &	Study design or study content is not
Sakong (2023)	Sakong, H., Cho, Y., & Seo, T. (2023). High or low volume beetroot juice	Study design or study content is not
Sandbakk (2015)	Sandbakk, S., Sandbakk, O., Peacock, O., James, P., Welde, B., Stokes, K., Böhlke, N.,	Study design or study content is not
Sebastiá-Rico	Sebastiá-Rico, J., Cabeza-Melendre, D., Anderson, L., & Martínez-Sanz, J. M. (2023).	Study design or study content is not
Senefeld (2020)	Senefeld, J., Wiggins, C., Regimbal, R., Dominelli, P., Baker, S., & Joyner, M. (2020).	Study design or study content is not
Shannon (2022)	Shannon, O., Allen, J., Bescos, R., Burke, L., Clifford, T., Easton, C., Gonzalez, J.,	Study design or study content is not
Shannon (2021)	Shannon, O. M., Easton, C., Shepherd, A. I., Siervo, M., Bailey, S. J., & Clifford, T.	Study design or study content is not
Shepherd (2015)	Shepherd, A. I., Wilkerson, D. P., Dobson, L., Kelly, J., Winyard, P. G., Jones, A. M.,	Study design or study content is not
Sheridan (2022)	Sheridan, H., Parker, L., & Hammond, K. (2022). Dietary supplements for consideration	Study design or study content is not
Shu (2025)	Shu, M., Liang, J., Jo, Y., Eom, S., & Kim, C. (2025). Applications and Benefits of	Study design or study content is not
Siervo (2013)	Siervo, M., Lara, J., Ogbonmwan, I., & Mathers, J. (2013). Inorganic Nitrate and	Study design or study content is not
Silva Díaz (2026)	Silva Díaz, V., Castillo-Paredes, A., Iman Torres, A. J., Alves Vargas, A. E., Guzmán-	Study design or study content is not
Silva (2022)	Silva, K., Costa, B., Gomes, A., Saunders, B., & Mota, J. (2022). Factors that Moderate	Study design or study content is not
Silva (2023)	Silva, W., Machado, P., Mesquita, V., Andrade, E., Lima, R., & Pereira, L. (2023).	Study design or study content is not
Smith (2022)	Smith, E., McKay, A., Kuikman, M., Ackerman, K., Harris, R., Elliott-Sale, K.,	Study design or study content is not
Smith (2019)	Smith, K., Muggeridge, D. J., Easton, C., & Ross, M. D. (2019). An acute dose of	Study design or study content is not
Souza Filho (2026)	Souza Filho, C. A. de, Ramos, O. J. F. J., Soares, R. N., & Alvares, T. da S. (2026).	Study design or study content is not
Szot (2022)	Szot, M., Karpecka-Galka, E., Drózdz, R., & Fraczek, B. (2022). Can Nutrients and	Study design or study content is not
Taati (2026)	Taati, B., Badicu, G., & Vveinhardt, J. (2026). Therapeutic Potential and Mechanistic	Study design or study content is not
Tan (2023a)	Tan, R., Baranauskas, M., Karl, S., de Zevallos, J., Shei, R., Paris, H., Wiggins, C., &	Study design or study content is not
Tan (2022)	Tan, R., Cano, L., Lago-Rodríguez, Á., & Domínguez, R. (2022). The Effects of Dietary	Study design or study content is not
Tan (2025)	Tan, R., Lincoln, I., Paniagua, K., Foster, J., Wideen, L., Gerardo, R., Ornelas, N.,	Study design or study content is not
Tan (2023)	Tan, R., Merrill, C., Price, K., Karl, S., Kenney, R., Riley, A., Riley, C., Hammer, M., &	Study design or study content is not
Tan (2023)	Tan, R., Pennell, A., Karl, S. T., Cass, J. K., Go, K., Clifford, T., Bailey, S. J., & Perkins	Study design or study content is not
Tan (2018)	Tan, R., Wylie, L. J., Thompson, C., Blackwell, J. R., Bailey, S. J., Vanhatalo, A., &	Study design or study content is not
Tatlici (2021)	Tatlici, A. (2021). The effects of acute beetroot juice supplementation on lower and	Study design or study content is not
Tatlici (2019)	Tatlici, A., & Çakmakçi, O. (2019). The effects of acute dietary nitrate supplementation	Study design or study content is not
Thompson (2016)	Thompson, C., Vanhatalo, A., Jell, H., Fulford, J., Carter, J., Nyman, L., Bailey, S. J., &	Study design or study content is not
Thompson (2018)	Thompson, C., Vanhatalo, A., Kadach, S., Wylie, L. J., Fulford, J., Ferguson, S. K.,	Study design or study content is not
Thompson (2014)	Thompson, K. G., Turner, L., Prichard, J., Dodd, F., Kennedy, D. O., Haskell, C.,	Study design or study content is not
Tillin (2018)	Tillin, N. A., Moudy, S., Nourse, K. M., & Tyler, C. J. (2018). Nitrate Supplement	Study design or study content is not
Vaughan (2016)	Vaughan, R. A., Gannon, N. P., & Carriker, C. R. (2016). Nitrate-containing beetroot	Study design or study content is not
Vicente-Salar	Vicente-Salar, N., Fuster-Muñoz, E., & Martínez-Rodríguez, A. (2022). Nutritional	Study design or study content is not
Volpe (2013)	Volpe, S. (2013). Does Beetroot Juice Really Help With Endurance Performance?	Study design or study content is not
Waldron (2018)	Waldron, M., Waldron, L., Lawlor, C., Gray, A., & Highton, J. (2018). Beetroot	Study design or study content is not
Wang (2025)	Wang, L., Zhao, R., Yan, Y., Zhang, H., Yan, R., Zhu, Y., Han, Z., Qu, Y., Wang, R., Li,	Study design or study content is not
Wei (2025)	Wei, C., Vanhatalo, A., Black, M., Rajaram, R., Massey, G., & Jones, A. (2025). Dose-	Study design or study content is not
Weitzberg (2011)	Weitzberg, E., & Lundberg, J. (2011). Dietary nitrate—A slow train coming. JOURNAL	Study design or study content is not
Wilkerson (2012)	Wilkerson, D. P., Hayward, G. M., Bailey, S. J., Vanhatalo, A., Blackwell, J. R., & Jones,	Study design or study content is not
Woessner (2018)	Woessner, M., McIlvenna, L., de Zevallos, J., Neil, C., & Allen, J. (2018). Dietary nitrate	Study design or study content is not
Woessner (2016)	Woessner, M., Smoliga, J. M., Tarzia, B., Stabler, T., Van Bruggen, M., & Allen, J. D.	Study design or study content is not
Wong (2024)	Wong, T. H., Sim, R., Sim, A., & Burns, S. F. (2024). Effects of an Isotonic Beetroot	Study design or study content is not
Wong (2021)	Wong, T., Sim, A., & Burns, S. (2021). The Effect of Beetroot Ingestion on High-	Study design or study content is not
Wylie (2016)	Wylie, L., Bailey, S., Kelly, J., Blackwell, J., Vanhatalo, A., & Jones, A. (2016).	Study design or study content is not
Wylie (2013)	Wylie, L., Kelly, J., Bailey, S., Blackwell, J., Skiba, P., Winyard, P., Jeukendrup, A.,	Study design or study content is not
Yang (2026)	Yang, X., Lu, Y., Lee, S. K., Xu, H., Chang, H., Liu, Q., & Quan, H. (2026). Beetroot	Study design or study content is not
Yang (2025)	Yang, X., Lu, Y., Xu, H., Liu, Q., Yun, D., Moon, Y., Quan, H., & Lee, S. (2025).	Study design or study content is not
Zafeiridis (2019)	Zafeiridis, A., Triantafyllou, A., Papadopoulos, S., Koletsos, N., Touplikioti, P.,	Study design or study content is not
Zamani (2021)	Zamani, H., de Joode, M., Hossein, I., Henckens, N., Guggeis, M., Berends, J., de Kok,	Study design or study content is not
Zart (2025)	Zart, S., & Fröhlich, M. (2025). Ergogenic effects of supplement combinations on	Study design or study content is not
Zoughaib (2024)	Zoughaib, W., Fry, M., Singhal, A., & Coggan, A. (2024). Beetroot juice	Study design or study content is not
**Non-original research (n = 11)**		
Afrisham (2023)	Afrisham, R., Farrokhi, V., Ghanavati, M., Asbaghi, O., Mohammadi, S., Mohammadian,	Non-original research
Cai (2026)	Cai, C., Huang, P., Cheng, X., & Zhou, J. (2026). Effectiveness of beetroot juice on	Non-original research
Christensen (2017)	Christensen, P., Shirai, Y., Ritz, C., & Nordsborg, N. (2017). Caffeine and Bicarbonate	Non-original research
Clements (2014)	Clements, W., Lee, S., & Bloomer, R. (2014). Nitrate Ingestion: A Review of the Health	Non-original research

Coutiño Díaz	Coutiño Díaz, M., Prieto Martínez, A., Zare, R., Ali Redha, A., & Forbes, S. C. (2025).	Non-original research
Domínguez (2017)	Domínguez, R., Cuenca, E., Maté-Muñoz, J., García-Fernández, P., Serra-Paya, N.,	Non-original research
Hlinsky (2020)	Hlinsky, T., Kumstát, M., & Vajda, P. (2020). Effects of Dietary Nitrates on Time Trial	Non-original research
Jones (2014)	Jones, A. (2014). Influence of dietary nitrate on the physiological determinants of	Non-original research
Jones (2018)	Jones, A., Thompson, C., Wylie, L., & Vanhatalo, A. (2018). Dietary Nitrate and	Non-original research
Poulios (2024)	Poulios, A., Papanikolaou, K., Draganidis, D., Tsimeas, P., Chatzinikolaou, A.,	Non-original research
Wickham (2019)	Wickham, K. A., & Spriet, L. L. (2019). No longer beeting around the bush: A review of	Non-original research
**Incomplete data (n = 7)**		
Harlow (2024)	Harlow, J., Blodgett, K., Stedman, J., & Pojednic, R. (2024). Dietary Supplementation on	Incomplete data
Montalvo-Alonso	Montalvo-Alonso, J., del Val-Manzano, M., Ferragut, C., Valadés, D., López-Samanes,	Incomplete data
Rohde (2018)	Rohde, A. (2018). The Effect Of Nitrate Supplementation Through Powdered Beetroot	Incomplete data
Salem (2026)	Salem, A., Ammar, A., Kerkeni, M., Boujelbane, M. A., Uyar, A. M., Köbel, L. M.,	Incomplete data
Serra-Payá (2021)	Serra-Payá, N., Garnacho-Castaño, M., Sánchez-Nuño, S., Albesa-Albiol, L., Girabent-	Incomplete data
Tan (2023b)	Tan, R., Baranauskas, M., Karl, S., de Zevallos, J., Shei, R., Paris, H., Wiggins, C., &	Incomplete data
Wylie (2013)	Wylie, L. J., Mohr, M., Krustrup, P., Jackman, S. R., Ermıdis, G., Kelly, J., Black, M. I.,	Incomplete data
**Unable to access full text (n = 8)**		
Abdo (2022)	Abdo, E., Allam, M., Gomaa, M., Shaltout, O., & Mansour, H. (2022). Valorization of	Unable to access full text
Eroglu (2026)	Eroglu, M. N., Kose, B., Kolayis, İ. E., & Haberal, B. (2026). Correction: Acute beetroot	Unable to access full text
Fernandes (2021)	Fernandes, H. (2021). RETRACTED: Dietary and Ergogenic Supplementation to	Unable to access full text
Kim (2020)	Kim, J., & Kim, E. (2020). Nutritional Strategies to Optimize Performance and Recovery	Unable to access full text
Larsen (2024)	Larsen, S. (2024). Acute antioxidant supplementation and performance—Should this be	Unable to access full text
McIntosh (2026)	McIntosh, M. C., Mueller, B. J., Tiede, D. R., Anglin, D. A., Kontos, G. J. J., Plotkin, D.	Unable to access full text
Peeling (2018)	Peeling, P., Binnie, M., Goods, P., Sim, M., & Burke, L. (2018). Evidence-Based	Unable to access full text
Rodrigues (2026)	Rodrigues, M. Y., Takito, M. Y., Kafouri, G. A., Pires, R. S., Mello, F. G., Zare, R.,	Unable to access full text
**Grey literature and non-randomized studies (n = 26)**		
Berends (2019)	Berends, J., van den Berg, L., Guggeis, M., Henckens, N., Hossein, I., de Joode, M.,	Grey literature and non-randomized
Borucka (2015)	Borucka, A. (2015). The influence of beetroot juice on triathletes performance. ANNALS	Grey literature and non-randomized
Calella (2025)	Calella, P., Di Dio, M., Pelullo, C. P., Di Giuseppe, G., Sansone, V., Liguori, F., Di	Grey literature and non-randomized
Capper (2019)	Capper, T., West, D., Siervo, M., & Stevenson, E. (2019). The effects of incremental	Grey literature and non-randomized
Curry (2016)	Curry, B. H., Bond, V., Pemminati, S., Gorantla, V. R., Volkova, Y. A., Kadur, K., &	Grey literature and non-randomized
Earnest (2019)	Earnest, C., Rothschild, J., Harnish, C., & Naderi, A. (2019). Metabolic adaptations to	Grey literature and non-randomized
Farzana (2026)	Farzana, H., Vigriawan, G., Santini, A., & Nurkolis, F. (2026). From Macronutrients to	Grey literature and non-randomized
Gallardo (2019)	Gallardo, E., & Coggan, A. (2019). What Is in Your Beet Juice? Nitrate and Nitrite	Grey literature and non-randomized
Giampaoli (2023)	Giampaoli, O., Ieno, C., Sciubba, F., Spagnoli, M., Miccheli, A., Tomassini, A., Aureli,	Grey literature and non-randomized
Holloway (2017)	Holloway, G. (2017). Nutrition and Training Influences on the Regulation of	Grey literature and non-randomized
Jurga (2024)	Jurga, J., Samborowska, E., Zielinski, J., & Olek, R. A. (2024). Effects of Acute Beetroot	Grey literature and non-randomized
Kozlowska (2020)	Kozlowska, L., Mizera, O., Gromadzinska, J., Janasik, B., Mikolajewska, K., Mróz, A.,	Grey literature and non-randomized
Lorei (2024)	Lorei, N., Ashley, R., Moberg, L., Butler, C., Seib, M. I., & Merseal, C. (2024). Beetroot	Grey literature and non-randomized
Lundberg (2013)	Lundberg, J. (2013). DIETARY NITRATE SUPPLEMENTATION AND EXERCISE	Grey literature and non-randomized
Matta (2026)	Matta, G., Edwards, A., Roelands, B., Hettinga, F., & Hurst, P. (2026). Physiological and	Grey literature and non-randomized
Mayra (2019)	Mayra, S., Johnston, C., & Sweazea, K. (2019). High-nitrate salad increased plasma	Grey literature and non-randomized
Miller (2022)	Miller, G., Nesbit, B., Kim-Shapiro, D., Basu, S., & Berry, M. (2022). Effect of Vitamin	Grey literature and non-randomized
Miraftabi (2021)	Miraftabi, H., Avazpoor, Z., Berjisian, E., Sarshin, A., Rezaei, S., Domínguez, R., Reale,	Grey literature and non-randomized
Nirmal (2023)	Nirmal, S., Olatunde, O., Medhe, S., Vitti, S., Khemtong, C., & Nirmal, N. (2023).	Grey literature and non-randomized
Ormsbee (2014)	Ormsbee, M., Bach, C., & Baur, D. (2014). Pre-Exercise Nutrition: The Role of	Grey literature and non-randomized
Patrician (2018)	Patrician, A., Engan, H., Lundsten, D., Grote, L., Vigetun-Haughey, H., & Schagatay, E.	Grey literature and non-randomized
Patrician (2017)	Patrician, A., & Schagatay, E. (2017). Dietary nitrate enhances arterial oxygen saturation	Grey literature and non-randomized
Poole (2022)	Poole, D., Ferguson, S., Musch, T., & Porcelli, S. (2022). Role of nitric oxide in	Grey literature and non-randomized
Reid (2016)	Reid, M. (2016). Redox interventions to increase exercise performance. JOURNAL OF	Grey literature and non-randomized
Stander (2021)	Stander, Z., Luies, L., van Reenen, M., Howatson, G., Keane, K., Clifford, T., Stevenson,	Grey literature and non-randomized
Van De Walle	Van De Walle, G., & Vukovich, M. (2018). THE EFFECT OF NITRATE	Grey literature and non-randomized
**Modified PEDro scale <16 and studies with missing data (n = 10)**		
Bender (2018)	Bender, D., Townsend, J., Vantrease, W., Marshall, A., Henry, R., Heffington, S., &	Modified PEDro scale <16 and
Berlanga (2023)	Berlanga, L. A., Lopez-Samanes, A., Martin-Lopez, J., de la Cruz, R. M., Garces-Rimon,	Modified PEDro scale <16 and
Burleigh (2020a)	Burleigh, M., Sculthorpe, N., Henriquez, F., & Easton, C. (2020a). Nitrate-rich Beetroot	Modified PEDro scale <16 and
Clifford (2016)	Clifford, T., Bell, O., West, D., Howatson, G., & Stevenson, E. (2016). The effects of	Modified PEDro scale <16 and
Clifford (2017)	Clifford, T., Bell, O., West, D., Howatson, G., & Stevenson, E. (2017). Antioxidant-rich	Modified PEDro scale <16 and
Clifford (2016a)	Clifford, T., Berntzen, B., Davison, G., West, D., Howatson, G., & Stevenson, E.	Modified PEDro scale <16 and
Clifford (2016b)	Clifford, T., Berntzen, B., Davison, G., West, D., Howatson, G., & Stevenson, E.	Modified PEDro scale <16 and
de Oliveira (2018)	de Oliveira, G., do Nascimento, L., Volino-Souza, M., Mesquita, J., & Alvares, T.	Modified PEDro scale <16 and
de Oliveira (2020)	de Oliveira, G., do Nascimento, L., Volino-Souza, M., Vellozo, O., & Alvares, T. (2020).	Modified PEDro scale <16 and
Esen (2024)	Esen, O., Fox, J., Karayigit, R., & Walshe, I. (2024). Acute Beetroot Juice	Modified PEDro scale <16 and
**Inclusion of populations not meeting criteria (n = 107)**		
Abreu (2023)	Abreu, R., Oliveira, C., Costa, J., Brito, J., & Teixeira, V. (2023). Effects of dietary	Inclusion of populations not
Adji (2022)	Adji, F. R., Sofro, Z. M., & Hapsari, M. (2022). The effect of beetroot juice (Beta	Inclusion of populations not
Ahmadpour (2024)	Ahmadpour, A., Fashi, M., & Hemmatinafar, M. (2024). Consuming Beetroot Juice	Inclusion of populations not
Alsharif (2023)	Alsharif, N., Clifford, T., Alhebshi, A., Rowland, S., & Bailey, S. (2023). Effects of	Inclusion of populations not
Alvares (2022)	Alvares, T. S., Oliveira, G. V. de, Volino-Souza, M., Conte-Junior, C. A., & Murias, J.	Inclusion of populations not
Alvares (2025)	Alvares, T. S., Pinheiro, V., Proctor, D. N., Conte Junior, C. A., & Nogueira Soares, R.	Inclusion of populations not
Amirpoor (2022)	Amirpoor, A., Zavar, R., Amerizadeh, A., Asgary, S., Moradi, S., Farzaei, M., Masoumi,	Inclusion of populations not

Ammar (2026)	Ammar, A., Salem, A., Kerkeni, M., Abril-Mera, T., Batalik, L., Toral-Noristz, M.,	Inclusion of populations not
Anderson (2022)	Anderson, O., Martinez-Ferran, M., Lorenzo-Calvo, J., Jiménez, S., & Pareja-Galeano,	Inclusion of populations not
Arazi (2021)	Arazi, H., & Eghbali, E. (2021). Possible Effects of Beetroot Supplementation on	Inclusion of populations not
Arnaoutis (2024)	Arnaoutis, G. (2024). Navigating Nutritional Challenges on the Way to Maximum	Inclusion of populations not
Bahrami (2021)	Bahrami, L., Arabi, S., Feizy, Z., & Rezvani, R. (2021). The effect of beetroot inorganic	Inclusion of populations not
Baiao (2019)	Baiao, D., d'El-Rei, J., Alves, G., Neves, M., Perrone, D., Del Aguila, E., & Paschoalin,	Inclusion of populations not
Bakker (2015)	Bakker, E., Engan, H., Patrician, A., Schagatay, E., Karlsen, T., Wisløff, U., & Gaustad,	Inclusion of populations not
Ballmann (2019)	Ballmann, C., Martin, M., Rogers, R., & Williams, T. (2019). Effects of Acute Beetroot	Inclusion of populations not
Barlow (2018)	Barlow, M. J., Elia, A., Shannon, O. M., Zacharogianni, A., & Lodin-Sundstrom, A.	Inclusion of populations not
Barnard (2022)	Barnard, J., Roberts, S., Lastella, M., Aisbett, B., & Condo, D. (2022). The Impact of	Inclusion of populations not
Basaqr (2021)	Basaqr, R., Skleres, M., Jayswal, R., & Thomas, D. T. (2021). The effect of dietary	Inclusion of populations not
Behrens (2020)	Behrens, C. E. J., Ahmed, K., Ricart, K., Linder, B., Fernández, J., Bertrand, B., Patel, R.	Inclusion of populations not
Benjamim (2023)	Benjamim, C. J. R., Sousa, Y. B. A., Porto, A. A., de Moraes Pontes, Y. M., Tavares, S.	Inclusion of populations not
Beresewicz (2016)	Beresewicz, A., & Gajos-Draus, A. (2016). Enjoy your heart-beets. The role of dietary	Inclusion of populations not
Betteridge (2016)	Betteridge, S., Bescós, R., Martorell, M., Pons, A., Garnham, A. P., Stathis, C. C., &	Inclusion of populations not
Bibi (2026)	Bibi, J., Lei, Y., Ahmad, M., Kotwica-Mojzych, K., Glowacka, M., & Mojzych, M.	Inclusion of populations not
Blau (2023)	Blau, L., Gerber, J., Finkel, A., Lützow, M., Maassen, N., Röhrich, M., Hanff, E., Tsikas,	Inclusion of populations not
Bloomer (2011)	Bloomer, R. J., Farney, T. M., Trepanowski, J. F., McCarthy, C. G., & Canale, R. E.	Inclusion of populations not
Bondonno (2015)	Bondonno, C., Liu, A., Croft, K., Ward, N., Shinde, S., Moodley, Y., Lundberg, J.,	Inclusion of populations not
Bonilla (2021)	Bonilla, D., Pérez-Idárraga, A., Odriozola-Martínez, A., & Kreider, R. (2021). The 4R's	Inclusion of populations not
Bonilla Ocampo	Bonilla Ocampo, D. A., Paipilla, A. F., Marín, E., Vargas-Molina, S., Petro, J. L., &	Inclusion of populations not
Braakhuis (2015)	Braakhuis, A., & Hopkins, W. (2015). Impact of Dietary Antioxidants on Sport	Inclusion of populations not
Brennan (2026)	Brennan, S. N., Foster, J. M., Gerardo, R. T., Molnar, T. J., Tran, R., Sottile, C. B.,	Inclusion of populations not
Brzezinska-Rojek	Brzezinska-Rojek, J., Sagatovych, S., Malinowska, P., Gadaj, K., Prokopowicz, M., &	Inclusion of populations not
Burke (2017)	Burke, L. (2017). Practical Issues in Evidence-Based Use of Performance Supplements:	Inclusion of populations not
Burke (2026)	Burke, L., Cox, G., Desbrow, B., Minehan, M., Morabito, A., Shaw, G., & Slater, G.	Inclusion of populations not
Burke (2021)	Burke, L., Hall, R., Heikura, I., Ross, M., Tee, N., Kent, G., Whitfield, J., Forbes, S.,	Inclusion of populations not
Burke (2018)	Burke, L., & Peeling, P. (2018). Methodologies for Investigating Performance Changes	Inclusion of populations not
Burleigh (2020b)	Burleigh, M., Sculthorpe, N., Henriquez, F., & Easton, C. (2020b). Nitrate-rich beetroot	Inclusion of populations not
Calvo (2020)	Calvo, J., Alorda-Capo, F., Pareja-Galeano, H., & Jiménez, S. (2020). Influence of	Inclusion of populations not
Campos (2018)	Campos, H., Drummond, L., Rodrigues, Q., Machado, F., Pires, W., Wanner, S., &	Inclusion of populations not
Casazza (2018)	Casazza, G., Tovar, A., Richardson, C., Cortez, A., & Davis, B. (2018). Energy	Inclusion of populations not
Chen (2024)	Chen, M., Chang, S., Xu, Y., Guo, H., & Liu, J. (2024). Dietary Beetroot Juice—Effects	Inclusion of populations not
Cherappurath	Cherappurath, N., Navaf, M., Yildiz, M., Ceylan, H., Thoompenthodi, M., Elayaraja, M.,	Inclusion of populations not
Choi (2026)	Choi, J.-H., Lee, E., Park, H.-Y., Jung, W.-S., Woo, S., Kim, S. Y., Zheng, Y., Park, S.	Inclusion of populations not
Cholewa (2019)	Cholewa, J., Trexler, E., Lima-Soares, F., de Araújo Pessôa, K., Sousa-Silva, R., Santos,	Inclusion of populations not
Coggan (2018)	Coggan, A. R., Broadstreet, S. R., Mikhalkova, D., Bole, I., Leibowitz, J. L.,	Inclusion of populations not
Costa (2022)	Costa, M., Toscano, L., Toscano, L., Luna, V., Torres, R., Silva, J., & Silva, A. (2022).	Inclusion of populations not
da Silva (2026)	da Silva, L. S. L., Gonçalves, L. D. S., Junior, M. F. T., Sousa, Y. B. A., Rebelo, M. A.,	Inclusion of populations not
Eggebeen (2016)	Eggebeen, J., Kim-Shapiro, D. B., Haykowsky, M., Morgan, T. M., Basu, S., Brubaker,	Inclusion of populations not
Fejes (2024)	Fejes, R., Pilat, N., Lutnik, M., Weisshaar, S., Weijler, A. M., Krüger, K., Draxler, A.,	Inclusion of populations not
Ferrada-Contreras	Ferrada-Contreras, E., Bonomini-Gnutzmann, R., Jorquera-Aguilera, C., Kuthe, N., Peña-	Inclusion of populations not
Ferreira (2011)	Ferreira, L., & Behnke, B. (2011). A toast to health and performance! Beetroot juice	Inclusion of populations not
Fiddler (2017)	Fiddler, R., & Lindel, N. (2017). No Effect of Acute Beetroot Juice Supplementation on	Inclusion of populations not
Finkel (2018)	Finkel, A., Röhrich, M., Maassen, N., Lützow, M., Blau, L., Hanff, E., Tsikas, D., &	Inclusion of populations not
Flanagan (2016)	Flanagan, S. D., Looney, D. P., Miller, M. J. S., DuPont, W. H., Pryor, L., Creighton, B.	Inclusion of populations not
Flueck (2016)	Flueck, J., Bogdanova, A., Mettler, S., & Perret, C. (2016). Is beetroot juice more	Inclusion of populations not
Flueck (2019)	Flueck, J. L., Gallo, A., Moelijker, N., Bogdanov, N., Bogdanova, A., & Perret, C.	Inclusion of populations not
Friis (2017)	Friis, A. L., Steenholt, C. B., Løkke, A., & Hansen, M. (2017). Dietary beetroot juice—	Inclusion of populations not
Gambardella (2021)	Gambardella, J., Fiordelisi, A., Spigno, L., Boldrini, L., Lungonelli, G., Di Vaia, E.,	Inclusion of populations not
Gamonales (2022)	Gamonales, J. M., Rojas-Valverde, D., Muñoz-Jiménez, J., Serrano-Moreno, W., &	Inclusion of populations not
Gaos (2026)	Gaos, S., Matos-Duarte, M., Ortiz, S., Ramasco, D., Muñoz, A., Aguilar-Navarro, M.,	Inclusion of populations not
Gilchrist (2014)	Gilchrist, M., Winyard, P., Fulford, J., Anning, C., Shore, A., & Benjamin, N. (2014).	Inclusion of populations not
Hennis (2016)	Hennis, P. J., Mitchell, K., Gilbert-Kawai, E., Bountziouka, V., Wade, A., Feelisch, M.,	Inclusion of populations not
Hoon (2015)	Hoon, M. W., Fornusek, C., Chapman, P. G., & Johnson, N. A. (2015). The effect of	Inclusion of populations not
Hoon (2013)	Hoon, M. W., Johnson, N. A., Chapman, P. G., & Burke, L. M. (2013). The effect of	Inclusion of populations not
Huang (2023)	Huang, X., Zhang, Z., Wang, X., Wang, G., Wang, Y., Tang, K., & Gao, B. (2023).	Inclusion of populations not
Jayawardena (2025)	Jayawardena, R., Weerasinghe, K., Nanayakkara, I., Madhujith, T., Hills, A., &	Inclusion of populations not
Jodra (2020)	Jodra, P., Lago-Rodríguez, A., Sánchez-Oliver, A., López-Samanes, A., Pérez-López, A.,	Inclusion of populations not
Kelly (2013)	Kelly, J., Fulford, J., Vanhatalo, A., Blackwell, J. R., French, O., Bailey, S. J., Gilchrist,	Inclusion of populations not
Kerley (2015)	Kerley, C., Cahill, K., Bolger, K., McGowan, A., Burke, C., Faul, J., & Cormican, L.	Inclusion of populations not
Kerley (2018)	Kerley, C., Dolan, E., James, P., & Cormican, L. (2018). Dietary nitrate lowers	Inclusion of populations not
Kerley (2019)	Kerley, C. P., James, P. E., McGowan, A., Faul, J., & Cormican, L. (2019). Dietary	Inclusion of populations not
Kuriakose (2016)	Kuriakose, R. K., Kukreja, R. C., & Xi, L. (2016). Potential Therapeutic Strategies for	Inclusion of populations not
Lee (2026)	Lee, E., Park, H., Sun, Y., Choi, J., Woo, S., Cho, S., Kim, S., Zheng, Y., Kim, S., &	Inclusion of populations not
Leong (2015)	Leong, P., Basham, J., Yong, T., Chazan, A., Finlay, P., Barnes, S., Bardin, P., &	Inclusion of populations not
McMahon (2017)	McMahon, N., Leveritt, M., & Pavey, T. (2017). The Effect of Dietary Nitrate	Inclusion of populations not
Murphy (2014)	Murphy, S., Witmer, C., Davis, S., & Sauers, E. (2014). Effects Of Beetroot Juice	Inclusion of populations not
Nowak (2025)	Nowak, A., Szymanska, A., Kwasniewska, M., Kochan, E., & Lipert, A. (2025). Beetroot	Inclusion of populations not
Nyberg (2021)	Nyberg, M., Christensen, P. M., Blackwell, J. R., Hostrup, M., Jones, A. M., & Bangsbo,	Inclusion of populations not
Olsson (2019)	Olsson, H., Al-Saadi, J., Oehler, D., Pergolizzi, J. J., & Magnusson, P. (2019).	Inclusion of populations not

Oue (2024)	Oue, A., Iimura, Y., Miyakoshi, Y., & Ota, M. (2024). Effect of Acute Dietary Nitrate	Inclusion of populations not
Pekas (2021)	Pekas, E. J., Wooden, T. K., Yadav, S. K., & Park, S.-Y. (2021). Body mass-normalized	Inclusion of populations not
Pinheiro (2025)	Pinheiro, V. D. S., Proctor, D. N., Nogueira Soares, R., & Alvares, T. S. (2025). Effect of	Inclusion of populations not
San Juan (2020)	San Juan, A. F., Dominguez, R., Lago-Rodríguez, Á., Montoya, J. J., Tan, R., & Bailey,	Inclusion of populations not
Shaltout (2017)	Shaltout, H. A., Eggebeen, J., Marsh, A. P., Brubaker, P. H., Laurienti, P. J., Burdette, J.	Inclusion of populations not
Shepherd (2015)	Shepherd, A. I., Gilchrist, M., Winyard, P. G., Jones, A. M., Hallmann, E., Kazimierczak,	Inclusion of populations not
Siervo (2016)	Siervo, M., Oggioni, C., Jakovljevic, D. G., Trenell, M., Mathers, J. C., Houghton, D.,	Inclusion of populations not
Tan (2024)	Tan, R., Cass, J. K., Lincoln, I. G., Wideen, L. E., Nicholl, M. J., Molnar, T. J., Gough,	Inclusion of populations not
Tan (2025)	Tan, R., Lincoln, I. G., Paniagua, K. K., Foster, J. M., Wideen, L. E., Gerardo, R. T.,	Inclusion of populations not
Tan (2024)	Tan, R., Merrill, C., Riley, C., Hammer, M., Kenney, R., Riley, A., Li, J., Zink, A., Karl,	Inclusion of populations not
Tan (2022)	Tan, R., Pennell, A., Price, K. M., Karl, S. T., Seekamp-Hicks, N. G., Paniagua, K. K.,	Inclusion of populations not
Tan (2023)	Tan, R., Price, K. M., Wideen, L. E., Lincoln, I. G., Karl, S. T., Seals, J. P., Paniagua, K.	Inclusion of populations not
Tan (2022)	Tan, R., Wylie, L. J., Wilkerson, D. P., Vanhatalo, A., & Jones, A. M. (2022). Effects of	Inclusion of populations not
Tanabe (2022)	Tanabe, Y., Fujii, N., & Suzuki, K. (2022). Dietary Supplementation for Attenuating	Inclusion of populations not
Thompson (2017)	Thompson, C., Wylie, L. J., Blackwell, J. R., Fulford, J., Black, M. I., Kelly, J.,	Inclusion of populations not
Tian (2025)	Tian, C., Jiang, Q., Han, M., Guo, L., Huang, R., Zhao, L., & Mao, S. (2025). Effects of	Inclusion of populations not
Turner (2022)	Turner, K. D., Kronemberger, A., Bae, D., Bock, J. M., Hughes, W. E., Ueda, K., Feider,	Inclusion of populations not
Vaccaro (2024)	Vaccaro, M. G., Innocenti, B., Cione, E., Gallelli, L., De Sarro, G., Bonilla, D. A., &	Inclusion of populations not
Van der Avoort	Van der Avoort, C., Van Loon, L., Hopman, M., & Verdijk, L. (2018). Increasing	Inclusion of populations not
Vicente-Salar	Vicente-Salar, N., Santos-Sánchez, G., & Roche, E. (2020). Nutritional Ergogenic Aids	Inclusion of populations not
Whitfield (2016)	Whitfield, J., Heigenhauser, G., Spriet, L., Holloway, G., & Tupling, A. (2016). Beetroot	Inclusion of populations not
Wickham (2019)	Wickham, K. A., McCarthy, D. G., Pereira, J. M., Cervone, D. T., Verdijk, L. B., van	Inclusion of populations not
Wickham (2018)	Wickham, K., McCarthy, D., Cervone, D., Verdijk, L., van Loon, L., & Spriet, L. (2018).	Inclusion of populations not
Wickham (2024)	Wickham, K., & Spriet, L. (2024). Food for thought: Physiological considerations for	Inclusion of populations not
Williams (2020)	Williams, T., Martin, M., Mintz, J., Rogers, R., & Ballmann, C. (2020). Effect of Acute	Inclusion of populations not
Wong (2022)	Wong, T. H., Sim, A., & Burns, S. F. (2022). The effects of nitrate ingestion on high-	Inclusion of populations not
Worley (2023)	Worley, M. L., Reed, E. L., Chapman, C. L., Kueck, P., Seymour, L., Fitts, T., Zazulak,	Inclusion of populations not
Yuschen (2024)	Yuschen, X., Choi, J.-H., Seo, J., Sun, Y., Lee, E., Kim, S.-W., & Park, H.-Y. (2024).	Inclusion of populations not
Zoughaib (2026)	Zoughaib, W. S., Fry, M. J., Singhal, A., Hoffman, R. L., & Coggan, A. R. (2026).	Inclusion of populations not
